# Hypoxia-inducible factor (HIF) prolyl hydroxylase inhibitors induce autophagy and have a protective effect in an *in-vitro* ischaemia model

**DOI:** 10.1038/s41598-020-58482-w

**Published:** 2020-01-31

**Authors:** Ayesha Singh, James W. Wilson, Christopher J. Schofield, Ruoli Chen

**Affiliations:** 1 Institute for Science and Technology in Medicine, School of Pharmacy, Keele University, Newcastle under Lyme, Staffordshire, ST5 5BG United Kingdom; 2 Chemistry Research Laboratory, Department of Chemistry, University of Oxford, Oxford, OX1 5JJ United Kingdom

**Keywords:** Neurochemistry, Cellular neuroscience, Molecular neuroscience

## Abstract

This study compared effects of five hypoxia-inducible factor (HIF) prolyl hydroxylases (PHD) inhibitors on PC12 cells and primary rat neurons following oxygen-glucose deprivation (OGD). At 100 µM, the PHD inhibitors did not cause cytotoxicity and apoptosis. MTT activity was only significantly reduced by FG4592 or Bayer 85–3934 in PC12 cells. The PHD inhibitors at 100 µM significantly increased the LC3-II/LC3-I expression ratio and downregulated p62 in PC12 cells, so did FG4592 (30 µM) and DMOG (100 µM) in neurons. HIF-1α was stabilised in PC12 cells by all the PHD inhibitors at 100 µM except for DMOG, which stabilised HIF-1α at 1 and 2 mM. In primary neurons, HIF-1α was stabilised by FG4592 (30 µM) and DMOG (100 µM). Pretreatment with the PHD inhibitors 24 hours followed by 24 hour reoxygenation prior to 6 hours OGD (0.3% O_2_) significantly reduced LDH release and increased MTT activity compared to vehicle (1% DMSO) pretreatment. In conclusion, the PHD inhibitors stabilise HIF-1α in normoxia, induce autophagy, and protect cells from a subsequent OGD insult. The new class of PHD inhibitors (FG4592, FG2216, GSK1278863, Bay85-3934) have the higher potency than DMOG. The interplay between autophagy, HIF stabilisation and neuroprotection in ischaemic stroke merits further investigation.

## Introduction

The hypoxia inducible transcription factors (HIF) are regulators of cellular responses to hypoxia in mammals. HIF mediated responses involve increases in the expression of multiple genes in a context dependent manner, including those associated with erythropoiesis and angiogenesis^1^. Under normoxic conditions, efficient catalysis by the ferrous iron and 2-oxoglutarate (2OG) dependent HIF prolyl hydroxylases (PHDs 1–3 in humans) promotes binding of HIF-α to the VHL (Von Hippel Lindau) tumour suppressor protein which is a targeting component of an E3 ligase complex, resulting in proteasomal degradation of HIF-α^2^. The action of a second 2-oxoglutarate (2OG) dependent HIF hydroxylase, the asparginyl hydroxylase, factor inhibiting HIF (FIH), regulates HIF activity by reducing its binding with histone acetyl transferases^2^. During hypoxia, PHD activity is reduced resulting in the stabilization and accumulation of HIF-1α in the cytoplasm, which then translocates to the nucleus forming the HIF transcription factors that upregulate expression of multiple genes^3^. The array of genes targeted by the HIF system makes it an appealing pharmacological target, in particular via PHD inhibition mediated HIF upregulation for treatment of diseases including anaemia, ischaemic stroke, and wound healing^4^. Currently, four PHD inhibitors i.e. FG4592 (Roxadustat) from FibroGen, GSK1278863 (Daprodustat) from GlaxoSmithKline, Bay85-3934 (Molidustat) from Bayer and AKB-6548 (Vadadustat) from Akebia are in clinical use or trials for anaemia treatment in patients with chronic kidney disease (CKD)^5,6^.

A number of HIF PHD inhibitors (e.g. GSK360A, FG4497, FG2216, DMOG, DFO) have been studied in stroke models either *in vivo* or *in vitro*, where these compounds showed neuroprotective effects following an ischaemic insult^7–12^. Nevertheless, the role of HIF in stroke pathophysiology remains debatable^13^. Neuronal specific HIF-1α knock out mice resulted in a worse neurological outcome and larger infarct volume following 30 min middle cerebral artery occlusion (MCAO)^14^; however, the neuronal specific HIF-1α knock out mice had better neurological outcomes after 75 min global ischaemia^15^. Single HIF1α or HIF-2α knock out mice had a similar infarct volume following MCAO compared to the respective wild type mice, possibly due to a mutual compensation^16^. HIF-1α/2α double knock out mice were observed to be significantly more impaired 72 hour after the MCAO suggesting HIF can be involved in functional recovery after cerebral ischaemia^16^. The effects of HIF on the ischaemic brain vary depending on the severity and/or duration of the stroke^13^. Whilst indirect induction of HIF, via genetic ablation of PHD1 or PHD2, reduced infarct volume and improved sensorimotor function following transient ischaemia^17,18^. The pharmacological inhibition of PHD2, and/or PHD1 could stimulate adaptations that provide protection against damage from ischaemic stroke^4,13^. A pharmacological approach for ischaemic cerebral tolerance is clinically appealing due to its non-invasive application. Currently, no available drug specifically inhibits a single PHD isoform, as such, researchers have mostly focused on generating inhibitors targeting PHD2, as it is proposed to be the most important PHD isoform in regulating HIF levels in normoxia^5^.

This study aimed to characterize effects of three clinical HIF-PHD inhibitors (FG4592, GSK1278863, Bay85-3934) alongside FG2216 (IOX3) and DMOG (dimethyloxalylglycine), a non-specific 2OG analogue, using an *in-vitro* oxygen-glucose deprivation (OGD) model. These chemicals activate the HIF signalling pathway in normoxia, with somewhat different effects^19^, which we considered could be reflected in specific effects on ischaemic neurons.

## Results

### PC 12 cells

#### Effects of PHD inhibitors on PC12 cell viabilities and apoptosis in normoxia

PHD inhibitors are widely used to mimic hypoxia *in vitro*, where they are initially dissolved and administered with DMSO. However, DMSO is a redox active small molecule and therefore could affect cell behaviour. In order to determine effects of DMSO on PC12 cells, a range of DMSO concentrations (0.5–10%) were administered to PC12 cells for 24 hours in normoxia. Both 5% and 10% DMSO produced significant reduction in MTT activity and increase in LDH release. Moreover, 2% DMSO produced a slight but significant reduction in MTT activity. The remaining concentrations of DMSO had no significant effects on MTT activity or LDH release compared to PC12 cultures into which no DMSO was added (Fig. S1). A concentration of 1% DMSO was therefore used in subsequent work to dissolve the PHD inhibitors.

**Figure 1:**
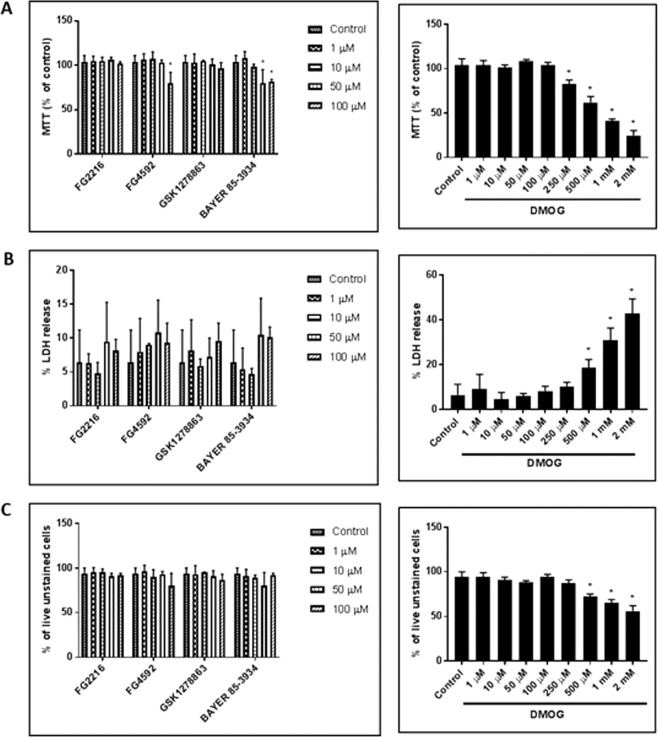
PC12 cells were cultured in normoxia (21% O_2_) for 24 hours with the PHD inhibitors (FG4592, FG2216, GSK1278863, Bay85-3934; concentration of 1, 10, 50, 100 µM) and DMOG (1, 10, 50, 100, 250, 500 µM, 1 and 2 mM). (**A**) MTT assays (n = 3) reveals reduction in mitochondrial activity (MTT release) in cells subjected to 100 µM of FG4592 and 50 & 100 µM of Bay85-3934 in comparison to 1% DMSO. The other inhibitors did not significantly affect mitochondrial activity. A significant reduction in MTT release was seen by 250, 500 µM, 1 and 2 mM of DMOG in comparison to 1% DMSO; (**B**). LDH assays (n = 3) revealed no significant changes in structural integrity (% LDH release) with FG4592, FG2216, GSK1278863, Bay85-3934 at all concentrations. DMOG increased % LDH release and resulted in significant cytotoxicity at concentration of 500 µM, 1 and 2 mM; (**C**). Trypan blue exclusion assay (n = 3) revealed no significant changes in % of live (trypan blue unstained cells) with FG4592, FG2216, GSK1278863, Bay85-3934 at all concentrations. DMOG significantly decreased % of live cells at concentration of 500 µM, 1 and 2 mM. Data were expressed as mean ± S.D. *Indicates P < 0.05 against 1% DMSO treated PC12 cells (Two-way ANOVA, Tukey’s *post-hoc* analysis).

**Figure 2:**
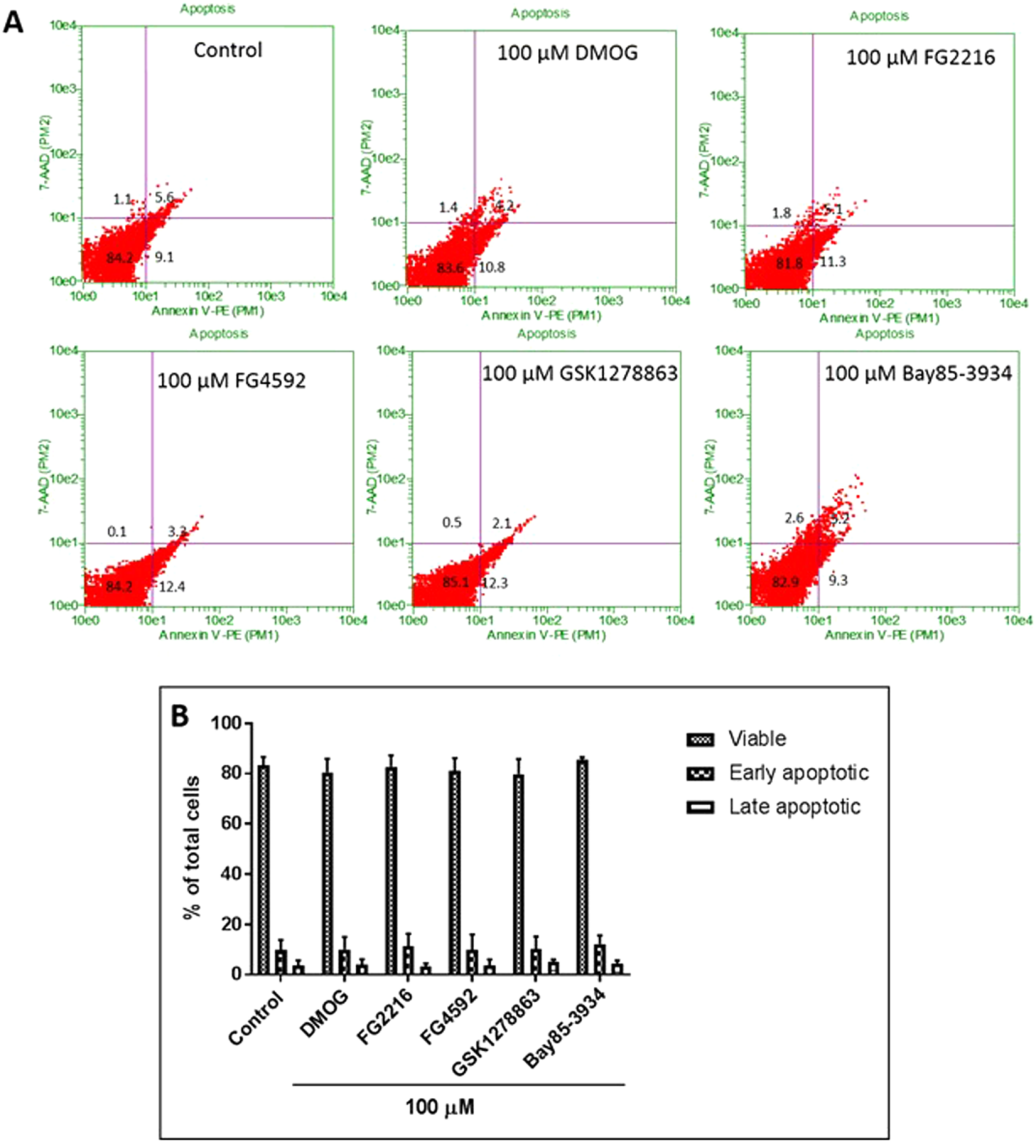
Annexin-V and 7-AAD FACS analysis of PC12 cells treated with HIF-PHD inhibitors (100 µM) in normoxia (21% O_2_) for 24 hours. (**A**) Representative dot plots of Annexin-V/7-AAD FACS analysis of cells treated with the vehicle (1% DMSO) and the inhibitors. Cells in lower left quadrant represent viable cells, cells in lower right quadrant represent early apoptosis and cells in upper right quadrant represent late apoptosis/necrosis; (**B**) The group data (n = 3) representing % of viable, early and late apoptotic cells. There was no significant different in any of the treatments groups compared to 1% DMSO treatment. Data were expressed as mean ± S.D.

We then evaluated the effects of varying concentrations (1, 10, 50, 100 µM) of the PHD inhibitors (FG2216, FG4592, GSK1278863 and Bay85-3934) and DMOG (1, 10, 50, 100, 250, 500 µM, 1 mM and 2 mM) in PC12 cell viabilities for 24 h in normoxia (21% O_2_), using MTT (mitochondrial activity), LDH (cytotoxicity) and trypan blue exclusion (cell viability) assays. Under 100 µM, the 4 “clinical” compounds did not manifest evidence for cytotoxicity (Fig. 1B), nor cause significant changes in the fraction of live cells (Fig. 1C). On the other hand, DMOG (500 µM, 1 mM and 2 mM) caused significant cytotoxicity and reduction in fraction of live cells. At high concentrations, two of the “clinical” compounds (FG4592, 100 µM and Bay 85–3934, 50 & 100 µM) and DMOG (250 µM, 500 µM, 1 mM and 2 mM) significantly reduced MTT activity, while the other compounds had no significant apparent effects on mitochondrial activity (Fig. 1A). Annexin-V (AV)/7-AAD FACS analysis was performed to determine whether the PHD inhibitors resulted in apoptosis. 24 hours oxygen deprivation was performed as a positive control (Fig. S2). The results revealed no significant changes in viable (AV^−^/7-AAD^−^), early apoptotic (AV^+^/7-AAD^−^) and late apoptotic/necrotic (AV^+^/7-AAD^+^) in cells treated with the PHD inhibitors (100 µM) in comparison to 1% DMSO (vehicle) (Fig. 2).

#### Effects of PHD inhibitors on autophagy

**Figure 3:**
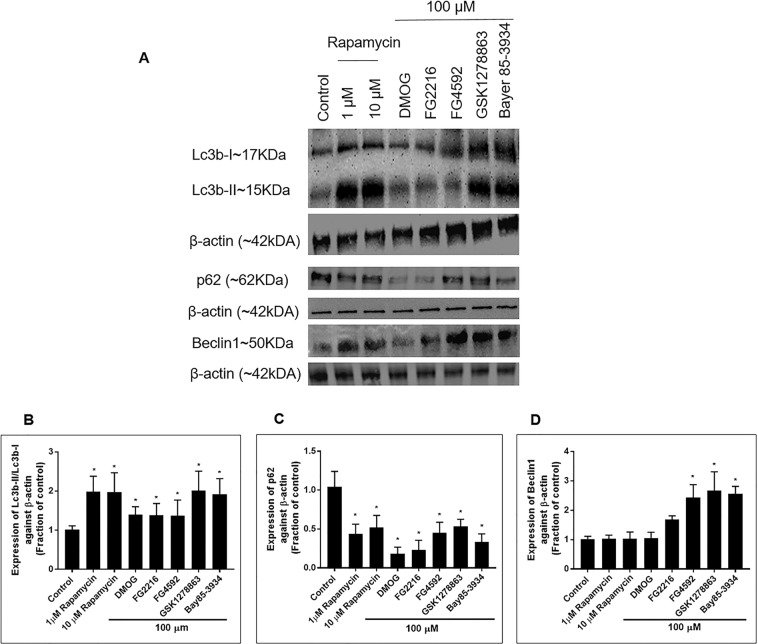
Immunoblot analysis of the protein expression of Lc3b-II, Lc3b-I, p62 and Beclin1 in PC12 cells treated with the indicated PHD inhibitors (100 µM) and rapamycin (1 & 10 µM) for 24 hours in normoxia. (**A**) Representative immunoblots of Lc3b-II, Lc3b-I, p62 and Beclin1 were shown alongside β-actin; (**B**) Normalised Lc3b-II/Lc3b-I ratio measured after 24 hours exposure to the indicated PHD inhibitors and rapamycin in normoxia (n = 3). Significant increase in the Lc3b-II/Lc3b-I ratio was seen with rapamycin (1 & 10 µM) and 100 µM of DMOG, FG2216, FG4592, GSK1278863 and Bay85-3934. GSK1278863 and Bay85-3934 had a similar effect on the Lc3b-II/Lc3b-I ratio as Rapamycin; (**C**) Expression of p62 measured after 24 hours exposure to the indicated PHD inhibitors and rapamycin in normoxia (n = 3). Significant reduction in p62 expression in comparison to vehicle (1% DMSO)-treated cells was seen with rapamycin (1 & 10 µM) and 100 µM of DMOG, FG2216, FG4592, GSK1278863 and Bay85-3934 treated cells; (**D**) Expression of Beclin1 measured after 24 hours exposure to the indicated PHD inhibitors and rapamycin in normoxia (n = 3). Significant upregulation of Beclin1 expression in comparison to vehicle (1% DMSO)-treated cells was seen in 100 µM of DMOG, FG2216, FG4592, GSK1278863 and Bay85-3934 treated cells, but was not in the Rapamycin treated cells. Data were expressed as mean ± S.D. *Indicated P < 0.05 against 1% DMSO treatment (Two-way ANOVA, Tukey’s post-hoc analysis).

We thereafter studied the effect of 24 hours treatment with the PHD inhibitors (100 µM) on LC3b (microtubule associated protein light-chain 3b), p62 (sequestosome-1, SQSTM1) and Beclin1 (BCN1) expression. Rapamycin, an established, autophagy inducer, was used as a positive control. There was a significant increase in LC3b-II/LC3b-I ratio and reduction in p62 in cells exposed to 1 and 10 µM of Rapamycin (Fig. 3). A significant increase in the LC3b-II/LC3b-I ratio was also seen in cells exposed to DMOG, FG2216 and FG4592, but at slightly lower levels compared to GSK1278863 and Bay85-3934. In comparison to 1% DMSO (vehicle) treated cells, there was significant downregulated in p62 in cells exposed to DMOG, FG2216, FG4592, GSK1278863 and Bay85-3934. A significant increase in Beclin1 was seen in cells exposed to FG4592, GSK1278863 and Bay85-3934 (100 μM) but not in cells exposed to Rapamycin (1, 10 μM) and DMOG (100 μM).

#### Effects of PHD inhibitors on HIF-1α protein expression

**Figure 4:**
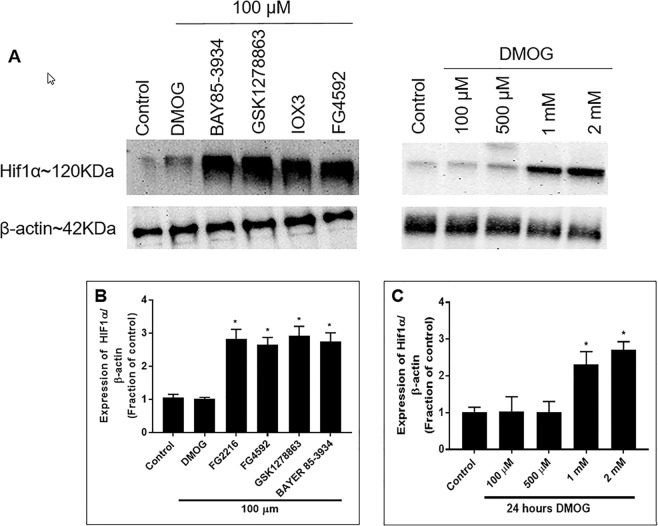
Immunoblot analysis of HIF-1α levels in PC12 cells treated with 100 µM of the indicated PHD inhibitors and DMOG (100 µM, 500 µM, 1 mM, 2 mM) for 24 hours in normoxia (21% O_2_). (**A**) Representative HIF-1α immunoblots were shown with those for β-actin; (**B**) Graph (n = 3) showed the normalised HIF-1α level measured at 24 hours after exposure to the indicated PHD inhibitors (100 µM) in normoxia. 24 hours incubation with 100 µM of FG2216, FG4592, GSK1278863 and Bay85-3934 significantly increased HIF-1α protein levels, while 100 µM DMOG did not change the HIF-1α proteinlevels; (**C**) Graph (n = 3) showing normalised HIF-1α level at 24 hours after exposure to DMOG (100 µM, 500 µM, 1 mM, 2 mM) in normoxia. Both 1 mM and 2 mM DMOG increased HIF-1α protein levels in normoxia. Data were expressed as mean ± S.D. *Indicated P < 0.05 against 1% DMSO treatment (Two-way ANOVA, Tukey’s *post-hoc* analysis).

Next, we investigated HIF-1α stabilisation at 24 hours PHD inhibitor treatment in normoxia. No significant stabilisation of HIF-1α was seen at concentrations of 1, 10 and 50 µM (data not shown). Significantly increased HIF-1α levels were observe in cells exposed to 100 µM of all the PHD inhibitors (FG2216, FG4592, GSK1278863 and Bayer 85-3934) (Fig. 4B), except for DMOG. Further studies demonstrated that HIF-1α was significantly stabilised in cells exposed to 1 mM and 2 mM of DMOG for 24 hours (Fig. 4C).

#### Hypoxia gene expression affected by the PHD inhibitors

**Figure 5:**
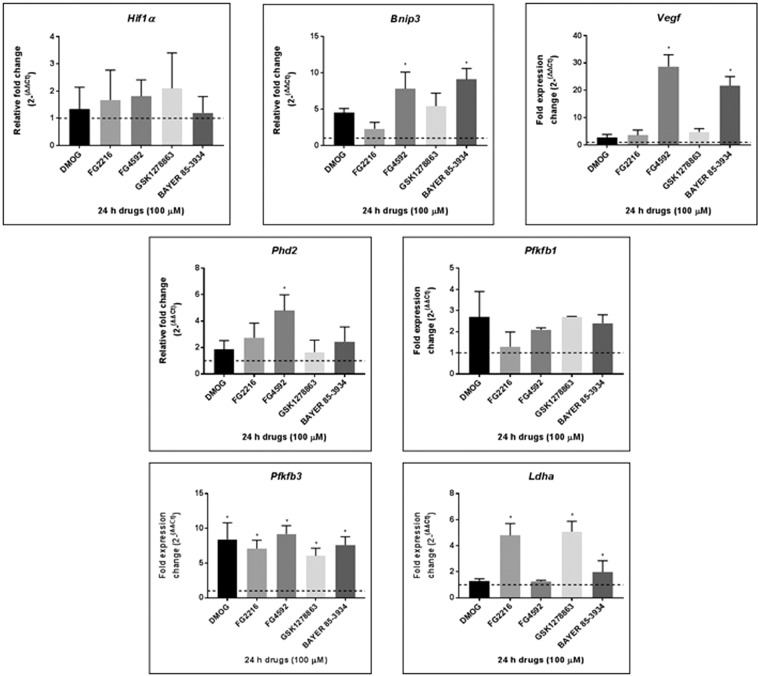
Effects of HIF PHD inhibitors on hypoxia gene expression. Expression of hypoxia genes, *HIF1α*, *Bnip3*, *Phd2*, *Vegf*, *Pfkfb1*, *Pfkfb3*, *Ldha*, after treatment with 100 µM PHD inhibitors in normoxia for 24 hours were normalised to *β-actin* levels. Significant upregulation of *Bnip3* and *Vegf* was seen with FG4592 and Bay85-3934. *Phd2* was significant upregulated by FG4592. *Pfkfb3* was significantly upregulated by DMOG, FG2216, FG492, GSK1278863, and Bay85-3934. *Ldha* was significantly upregulated by FG2216, GSK1278863 and Bay85-3934. *HIF1α* was not changed by any of the HIF PHD inhibitors. Each data point represented the mean and standard deviation of the relative fold change with respect to 1% DMSO-treated sample normalised to reference gene *β-actin* level. The dotted line represented basal gene expression. Statistical significance was indicated as *P < 0.05 (Two-way ANOVA, Tukey’s *post-hoc* analysis).

Using qRT-PCR, we then investigated whether the PHD inhibitors (at 100 µM) have an effect on hypoxia gene expressions (*Hif1α*, *Bnip3*, *Phd2*, *Vegf*, *Pfkfb1*, *Pfkfb3*, *and Ldha)* in PC12 cells (Fig. 5). Of all the genes studied, *Pfkfb3* was most consistently upregulated, i.e. by DMOG (~8.5 fold), FG2216 (~7 fold), FG4592 (~9 fold), GSK1278863 (~6 fold) and Bay85-3934 (~7.5 fold), while *Pfkfb1* expression was not significantly changed. Significant *Ldha* upregulation was observed with FG2216 (~5 fold), GSK1278863 (~5 fold) and Bay85-3934 (~2 fold). *Bnip3* was significantly upregulated by FG4592 and Bay85-3934 with fold changes of ∼8 and ∼9, respectively. *Phd2* was mildly upregulated by the PHD inhibitors, but significant fold change was seen by FG4592 (~5 fold). *Vegf* expression was upregulated in PC12 cells following 100 µM of FG4592 (~28 fold) and Bay85-3934 (~21 fold). *Hif1α* expression was not significantly changed by the PHD inhibitors.

#### Effects of preconditioning with PHD inhibitors and re-oxygenation prior to OGD in PC12 cells

We then studied whether pre-treatment with the PHD inhibitors followed by re-oxygenation was protective for PC12 cells against subsequent OGD insult. The effects of various concentrations (1, 10, 50 and 100 µM) were studied. PC12 cells were subjected to 24 hours PHD inhibitors treatment followed by a period of 24 hours reoxygenation prior to an OGD insult (6 hours, 0.3% O_2_). Six hours OGD (0.3% O_2_) led to about ~40% of cell death, of which ~90% were early apoptotic, ~10% were late apoptotic/necrotic, and caused significant increase of LDH release (24.3% ± 2.3% vs 6.4% ± 2.2%) and reduction in MTT activity (73.6% ± 12.3% vs 100%), compared to the normoxia control.

**Figure 6:**
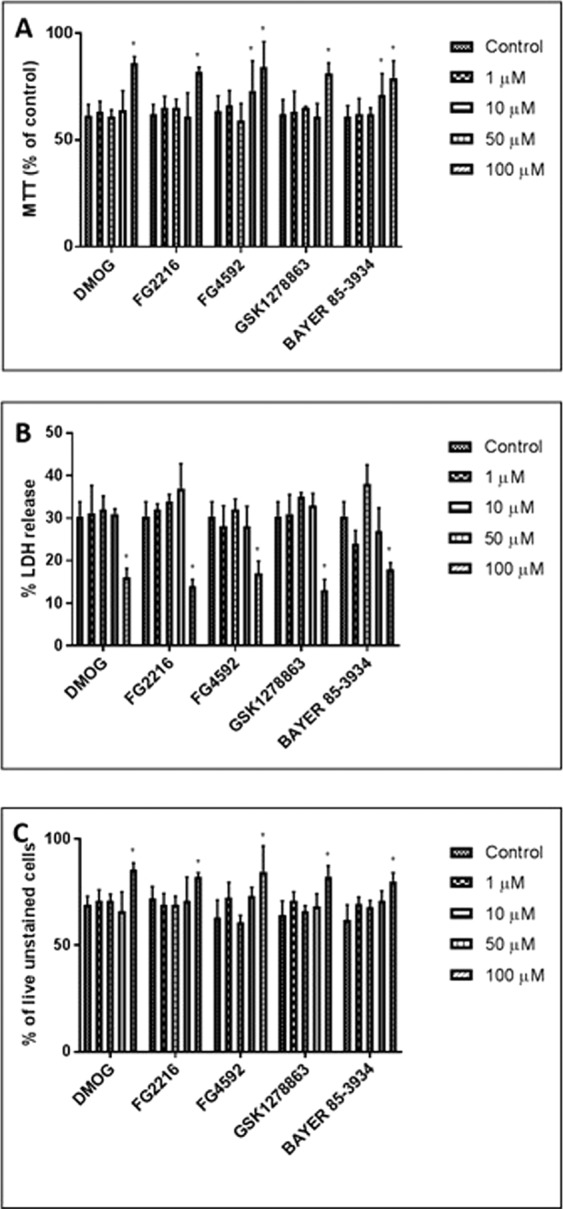
The effect of preconditioning with HIF-PHD inhibitors followed by 24 hours re-oxygenation and 6 hours OGD on PC12 cells. PC12 cells were exposed to the PHD inhibitors (1, 10, 50 and 100 µM) for 24 hours in normoxia (21% O_2_) followed by 24 hours re-oxygenation (21% O_2_) and subsequent 6 hours OGD insult (0.3% O_2_). (**A**) MTT assay (n = 3) showed greater mitochondrial activity after 6 hours OGD in cells preconditioned with 100 µM of DMOG, FG2216, or GSK1278863 and 50 & 100 µM of FG4592 or Bay 85-3934 compared to 1% DMSO pretreatment; (**B**) LDH assays (n = 3) revealed reduced LDH release in cells preconditioned with 100 µM of DMOG, FG2216, FG4592, GSK1278863, or Bay 85-3934 prior to 6 hours OGD compared to 1% DMSO pretreatment; (**C**) Trypan blue exclusion assays (n = 3) reveal increased live (unstained) cells in cells preconditioned with 100 µM of DMOG, FG2216, FG4592, GSK1278863, or Bay 85-3934 prior to 6 hours OGD compared to 1% DMSO pretreatment. Data were expressed as mean ± S.D. *Indicated P < 0.05 against 1% DMSO (Two-way ANOVA, Tukey’s *post-hoc* analysis).

**Figure 7:**
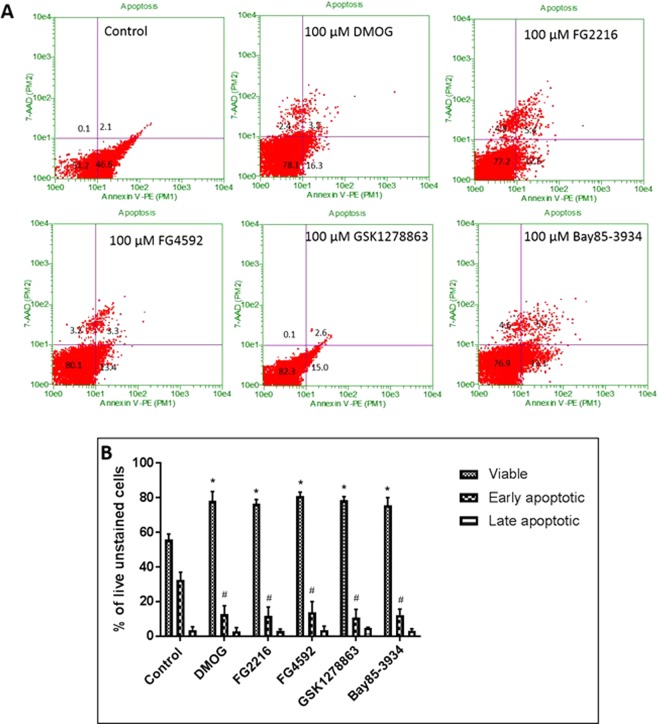
Annexin-V/7-AAD FACS analysis of PC12 cells preconditioned with the HIF PHD inhibitors followed by 24 hours re-oxygenation and 6 hours OGD. PC12 cells were exposed to PHD inhibitors (100 µM) for 24 hours in normoxia (21% O_2_), followed by 24 hours re-oxygenation (21% O_2_) and subsequent 6 hours OGD treatment (0.3% O_2_). (**A**) Representative dot plots of Annexin-V/7-AAD FACS analysis of cells with the PHD inhibitor pre-treatment and 24 hours re-oxygenation followed by 6 hours OGD. Cells in the lower left quadrant represent viable cells, cells in the lower right quadrant represent early apoptosis and cells in upper right quadrant represent late apoptosis/necrosis; (**B**) The group data (n = 3) representing % of viable, early and late apoptotic cells. Significant reductions in the fractions of early apoptotic cells were seen in cells preconditioned with 100 µM of DMOG, FG2216, FG4592, GSK1278863 and Bay 85-3934 in comparison to 1% DMSO-treated cells following 24 hours re-oxygenation and 6 hours OGD. Data were expressed as mean ± S.D. *Indicated significance of viable cells P < 0.05 against 1% DMSO. ^#^Indicated significance of apoptotic cells P < 0.05 against 1% DMSO (Two-way ANOVA, Tukey’s *post-hoc* analysis).

Pre-treatment with the PHD inhibitors at 100 µM for 24 hours followed by 24 hours of reoxygenation significantly reduced the cell death and LDH release caused by the 6 hours OGD (0.3% O_2_) compared to the vehicle (1% DMSO) pre-treated cells (Fig. 6). These compounds at 100 µM, as well as FG4592 and Bay85-3934 at 50 µM significantly increased the MTT activities in cells after the OGD (Fig. 6). FACS analysis (Fig. 7) of cells double stained with Annexin-V and 7-AAD revealed no significant difference in late apoptotic/necrotic (AV^+^/7-AAD^+^) cells between 1% DMSO (vehicle) and PHD inhibitors pre-treated groups. There was a significant reduction in early apoptotic cells when pre-treated with DMOG (15.7 ± 4.9%), FG2216 (11.8 ± 5.1%), FG4592 (16.9 ± 7.1%), GSK1278863 (10.7 ± 4.8%) and Bay85-3934 (12.3 ± 3.8%), in comparison to the 1% DMSO-treated cells (32.5 ± 4.5%). The pretreatment of PHD inhibitors increased significantly in cell viable (AV^−^/7-AAD^−^) to ~75–80% following the 6 hours OGD, compared to those pretreated by 1% DMSO (~55%).

### Primary rat neurons

#### Effects of PHD inhibitors on primary rat cortical neurons viabilities in normoxia

**Figure 8:**
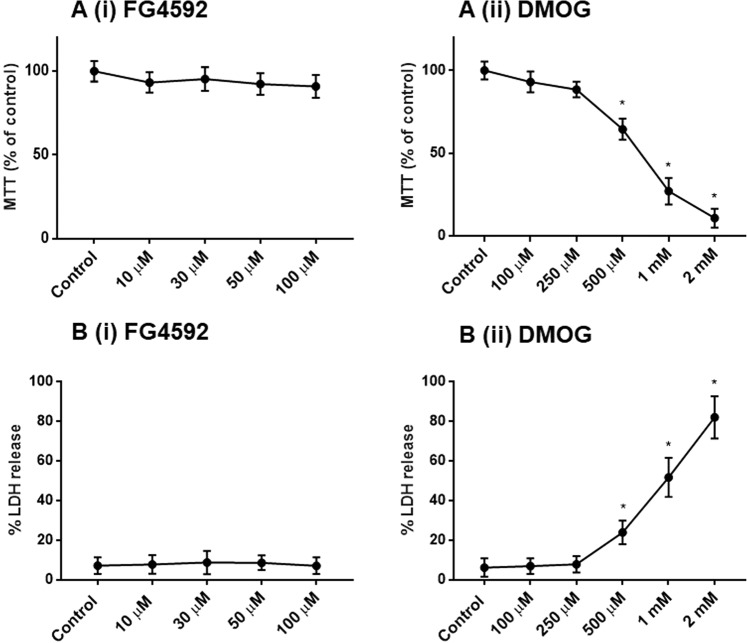
Effect of HIF-PHD inhibitors on primary rat neurons during normoxia. Primary rat cortical neurons were cultured in normoxia (21% O_2_) for 24 hours with control (1% DMSO), FG4592 (concentration of 10, 30 50, 100 µM) and DMOG (100, 250, 500 µM, 1 mM and 2 mM). (**A**) MTT assays (n = 3) reveals no significant changes in mitochondrial activity in primary neurons subjected to 10- 100 µM of FG4592 (i), while a significant reduction in MTT activity was seen 500 µM, 1 mM and 2 mM of DMOG (ii), in comparison to control (1% DMSO) treated cells; (**B**). LDH assays (n = 3) revealed no significant changes in LDH release (%) with FG4592 (10–100 µM)(i), while a significant increase in % LDH release was seen by 500 µM, 1 mM and 2 mM of DMOG (ii) in comparison to control (1% DMSO) treated neurons.

**Figure 9:**
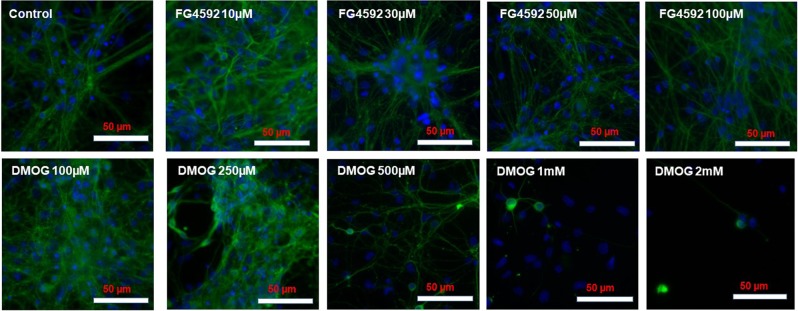
Representative double merged (FITC labelled neuron specific Tuj1 and DAPI stained nuclei) immunofluorescence images of primary rat neurons following FG4592 and DMOG treatment in normoxia. No morphological changes were observed with FG4592 treatment (10–100 µM) and DMOG (100, 250 µM), but DMOG (500 µM, 1 mM and 2 mM) treatment resulted in neurons with shorter and fewer neurites and reduced cell densities per microscopic field.

Similar to PC 12 cells, 1% DMSO did not cause significant changes in MTT activity and LDH release in primary neuron cultures (Fig. S3), and was used as the vehicle to dissolve FG4592 (10, 30, 50, 100 µM) and DMOG (100, 250, 500 µM, 1 mM and 2 mM) for studying their effects in primary neuron cultures. Since the 4 “clinical” compounds (FG4592, GSK1278863, Bay 85–3934, FG2216) had similar effects on the PC 12 cells, we used FG4592 as the representative to compare with DMOG (a non-specific 2-OG analogue) in primary neurons. Under 100 µM, neither FG4592 nor DMOG altered MTT activity, LDH release (Fig. 8A,B) and cell morphology (Fig. 9). DMOG at concentrations of 500 µM, 1 mM and 2 mM, however, caused significant LDH release and reduced MTT activities (Fig. 8A,B) as well as morphology changes in the neurons, such as shorter and fewer neurites, reduced nuclei densities per microscopic field (Fig. 9).

#### Effects of PHD inhibitors on autophagy

**Figure 10:**
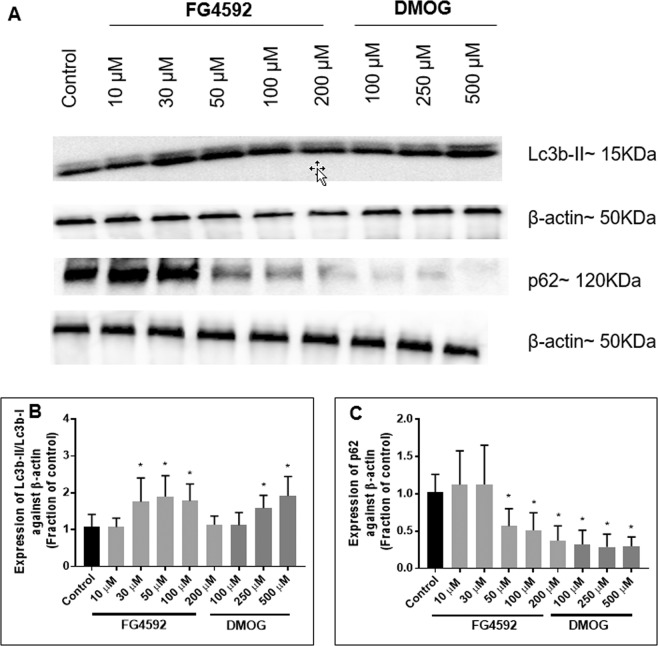
Effect of HIF-PHD inhibitors on the Lc3b-II/Lc3b-I and p62 expression in primary rat neurons. Immunoblot analysis of the Lc3b-II/Lc3b-I ratio and p62 in primary rat neurons treatment with 24 hours with control (1% DMSO), FG4592 (10, 30 50, 100 µM) and DMOG (100, 250 µM). (**A**) Representative Lc3b-II/Lc3b-I and p62 immunoblots alongside β-actin; (**B**) Normalised Lc3b-II/Lc3b-I ratio measured after 24 hours exposure to FG4592 or DMOG in normoxia (n = 3). Significant increase in the Lc3b-II/Lc3b-I ratio was seen with FG4592 (50, 100 µM) and DMOG (250, 500 µM) in comparison to control (1% DMSO-treated cells); (**C**) Expression of p62 measured after 24 hours exposure to FG4592 or DMOG in normoxia (n = 3). Significant reduction in p62 expression in comparison to control (1% DMSO-treated cells) was seen 50, 100 µM of FG4592 and 100, 250, 500 µM of DMOG treated cells. Error bars represent ± S.D. *Indicates P < 0.05 against control (1% DMSO treatment) (Two-way ANOVA, Tukey’s post-hoc analysis).

In the primary neurons, 24 hours treatment with FG4592 and DMOG resulted in a significant increase in Lc3b-II/Lc3b-I ratio [FG4592 (30, 50 and 100 µM) and DMOG (250 and 500 µM)], significant downregulation of p62 [FG4592 (50, 100 µM) and DMOG (100, 250 and 500 µM)] (Fig. 10). FG4592 (10 µM) did not result in significant changes in LC3b and p62 expression (Fig. 10).

#### Effects of PHD inhibitors on HIF-1α protein expression

**Figure 11:**
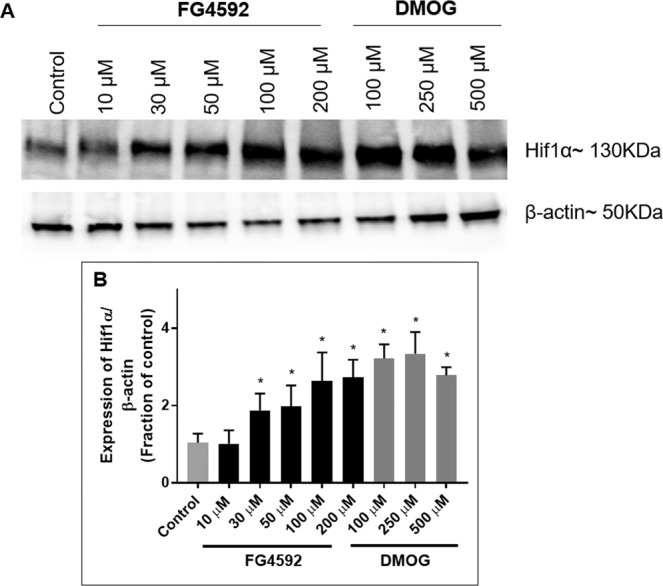
Effects of HIF-PHD inhibitors on HIF-1α levels in primary rat neurons. Immunoblot analysis of HIF-1α levels in primary cortical neurons treated with 24 hours with 1% DMSO, FG4592 (10, 30 50, 100 µM) and DMOG (100, 250 µM) in normoxia. (**A**) Representative HIF-1α immunoblots were shown with those for β-actin; (**B**) Graph (n = 3) showed the normalised HIF-1α level measured at 24 hours after exposure to FG4592 or DMOG in normoxia. FG4592 (30 µM onwards) and DMOG (100, 250 µM) significantly increased HIF-1α levels in primary rat neurons. Error bars represent ± S.D. *Indicates P < 0.05 against control (1% DMSO treatment) (Two-way ANOVA, Tukey’s *post-hoc* analysis).

Both FG4592 and DMOG stabilized HIF-1 at lower concentration following 24 hours incubation in primary rat neurons compared to PC 12 cells. In primary neurons, FG4592 (30, 50, 100 µM), and DMOG (100, 250, 500 µM) resulted in significant HIF-1 stabilisation (Fig. 11).

#### Effects of preconditioning with PHD inhibitors and re-oxygenation prior to OGD in primary rat neurons

**Figure 12:**
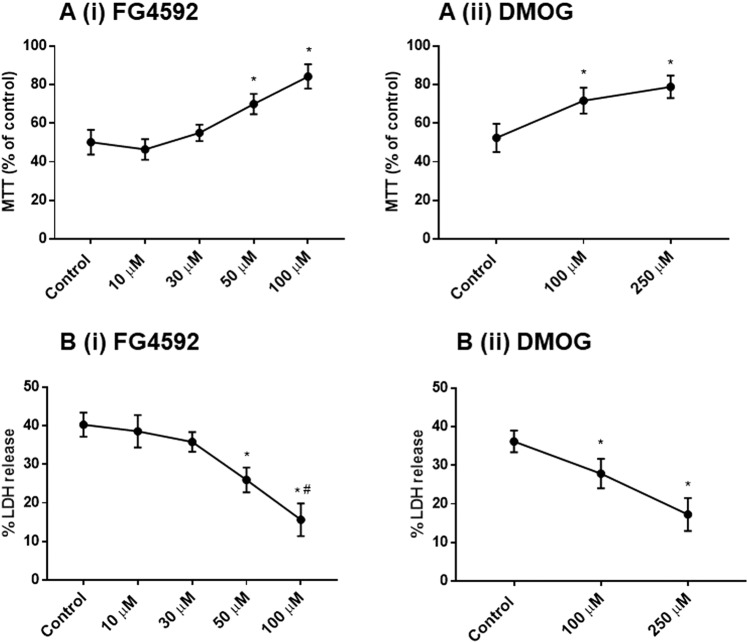
The effect of preconditioning with HIF-PHD inhibitors followed by 24 hours re-oxygenation and 6 hours OGD on primary rat neurons. Primary cortical rat neurons were exposed to 1% DMSO, FG4592 (10, 30 50, 100 µM) and DMOG (100, 250 µM) for 24 hours in normoxia (21% O_2_) followed by 24 hours re-oxygenation (21% O_2_) and subsequent 6 hours OGD insult (0.3% O_2_). (**A**) MTT assay (n = 3) showed greater mitochondrial activity after 6 h OGD in neurons preconditioned with FG4592 (50, 100 µM) (i) and DMOG (100, 250 µM) (ii) compared to 1% DMSO pretreatment; (**B**) LDH assays (n = 3) reveal reduced LDH release in cells preconditioned with FG4592 (50, 100 µM) (i) and DMOG (100, 250 µM) (ii) prior to 6 h OGD compared to 1% DMSO pretreatment.

**Figure 13:**
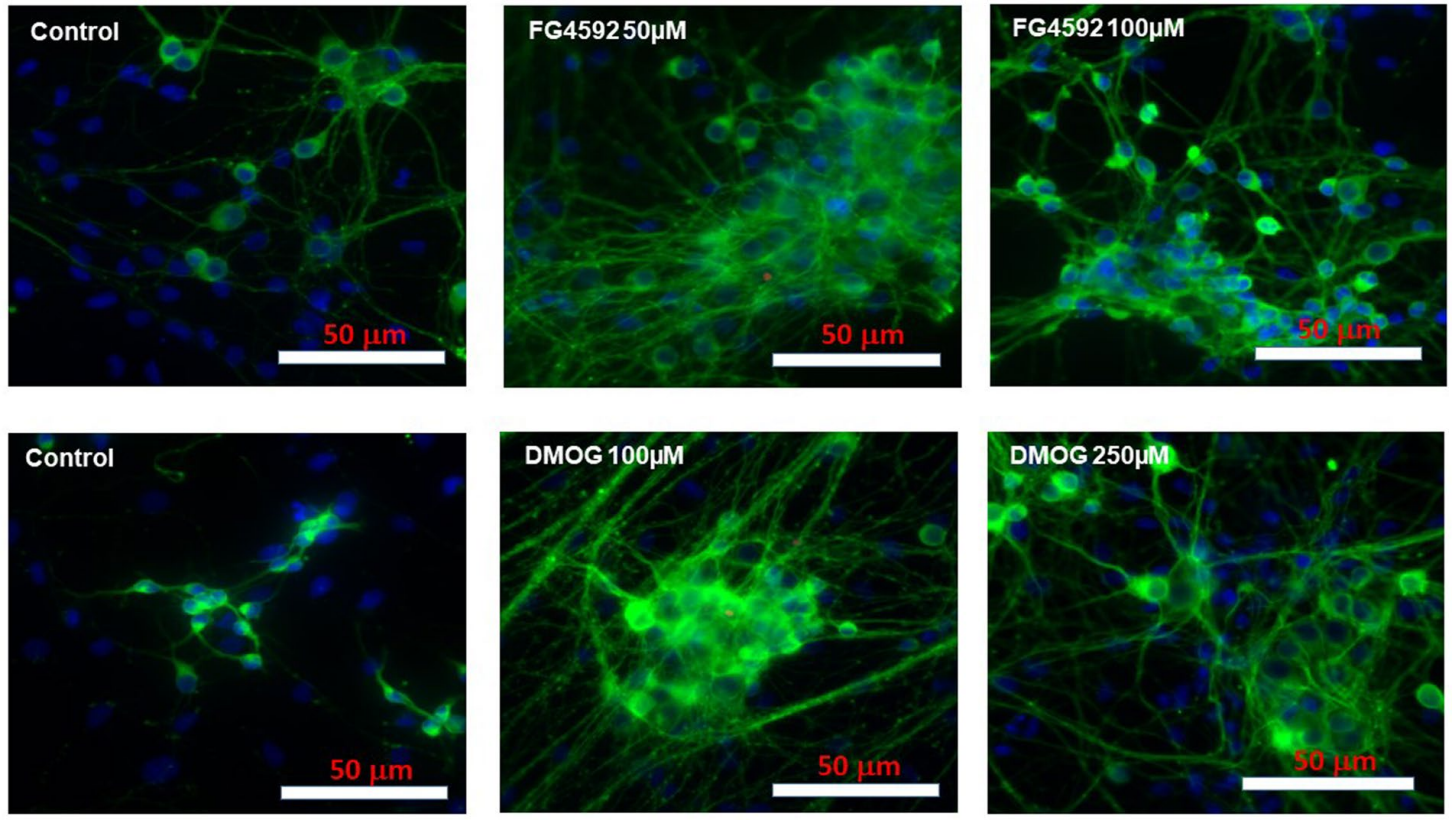
Representative double merged (FITC labelled neuron specific Tuj1 and DAPI stained nuclei) immunofluorescence images of primary rat neurons preconditioning with FG4592 and DMOG followed by 6 hours OGD. FG4592 (50, 100 µM) and DMOG (100, 250 µM) preconditioned neurons displayed longer and more neurites than those pretreated with 1% DMSO.

Pre-treatment with the FG4592 (50, 100 µM) and DMOG (100, 250 µM) for 24 hours followed by 24 hours of reoxygenation significantly increased the MTT activities in cells after the OGD and reduces LDH release caused by the 6 hours OGD (0.3% O_2_) compared to the vehicle (1% DMSO) pre-treated cells (Fig. 12A,B). FG4592 (50, 100 µM) and DMOG (100 and 250 µM) pre-treated cultures comprised of primary neurons (Tuj1^+^ cells) with longer and many neurites, compared to those pretreated with the vehicle (1% DMSO) (Fig. 13).

## Discussion

Stroke is the fourth main cause of mortality in the UK and the leading cause of adult disability, with cerebral ischaemia representing the most prevalent type of stroke (accounting for ~87% of cases)^20^. Over the past 30 years, various neuroprotective strategies for ischaemic stroke have failed to succeed in clinical trials due to the complex nature of stroke. Most of these strategies were aimed at targeting single molecules in the ischaemic cascade^21^. Recent focus has shifted towards targeting the brain’s own endogenous protective mechanism^22,23^. Our study investigated one such promising approach known as ischaemic tolerance^7,10,11^.

Our study focused on analysing the most recent clinically advanced small molecule PHD inhibitors at biologically safe concentrations. These PHD inhibitors are being pursued for treatment of anaemia^24^. We discovered that the PHD inhibitors stabilised HIF-1α in normoxia, and upregulated a number of known HIF target genes expression in PC12 cells. The PHD inhibitors induced autophagy, but not apoptosis in normoxia. Importantly, these PHD inhibitors protected the PC12 cells from OGD insults when given as preconditioning agents. Our studies were conducted using a PC12 cell model of ischaemia tolerance^25^, as PC12 cells have been extensively used in neurobiological studies as models of Alzheimer’s disease, Parkinson’s disease, Huntington’s disease and ischaemic stroke^25–27^. Since the 4 “clinical” PHD inhibitors had the similar effects on the PC12 cells, we then used FG4592 as a representative novel PHD inhibitor alongside with DMOG, a non-specific 2-OG analogue, in primary rat neurons, to validate the results obtained from the PC 12 cells. Both FG4592 and DMOG stabilized HIF-1α in the neurons at lower concentrations compared to PC12 cells; suggesting neurons are more sensitive to PHD inhibitors than PC12 cells. Similarly, both FG4592 and DMOG protected the neurons from OGD insults when given preconditioning.

At baseline, none of these compounds at 1–100 μM led to cytotoxicity (as assayed by LDH assays), cell death (as assayed by trypan blue exclusion assay) or apoptosis (as assayed by Annexin V/7-AAD staining) in PC 12 cells. MTT activities were unchanged by the compounds except with FG4592 (100 µM) and Bay 85–8934 (50 & 100 µM), which significantly decreased MTT activities. In primary neuron culture, no significant LDH release and MTT activity alteration was seen by FG4592 (100 µM) and DMOG (100 µM). On the other hand, DMOG (500 µM and above) resulted in significant LDH release and MTT activity reduction following 24 hours incubation in both PC 12 cells and primary neurons. MTT assay assesses mitochondrial catabolism and cell metabolic activity, which can be reduced in cells whilst retaining cell membrane integrity represented by LDH assay^28^.

Next, the role of the PHD inhibitors on three major autophagic marker, LC3, p62 and Beclin1 was studied using rapamycin as a positive control inducer of autophagy^29^. Beclin1 plays an important role in induction of autophagy. Upon induction of autophagy, cytosolic LC3-I is conjugated to phosphatidylethanolamine to form LC3-II, which is incorporated into the autophagosomal membrane. The p62 protein (sequestosome 1) binds to polyubiquitinated proteins through an ubiquitin-associated domain and combines with LC3-II through its LC3-interaction region domain for attachment to the autophagosome, which is finally degraded in autolysosome. In the process of autophagy, p62 is continuously degraded. Beclin1, LC3-II and p62 have been used to monitor autophagic activity in various studies^30^. Our results showed that both the PHD inhibitors (100 µM) and rapamycin (1, 10 µM) significantly increased LC3b-II/LC3b-I expression ratio. GSK1278863 (100 µM) and Bayer 85–3934 (100 µM) promoted LC3-II expression to a similar extent as rapamycin (10 µM). FG4592, FG2216 and DMOG induced LC3-II expression to a lesser extent, but still significant extent. A subsequent downregulation of p62 was seen in cells treated with rapamycin and the PHD inhibitors in comparison to the vehicle (1% DMSO) - treated cells indicating degradation by autolysosomes was initiated. Our results are consistent with a study by Duran *et al*.^31^ reporting inhibition of PHDs by DMOG for 5 hours in U2OS cells was sufficient to induce autophagy. Similarly, DMOG induced IC3-II accumulation and P62 degradation in HeLa cells^32^. In a rat model of CKD, DMOG treatment for 1 week significantly increased autophagy^33^. Li *et al*.^34^, reported that FG4592 induced autophagy in SH-SY5Y cells via LC3II/LC3I upregulation and p62 downregulation. The “clinical” PHD inhibitors significantly upregulated Beclin1 in the PC12 cells, which is line with the study by Lu *et al*.^35^ showed that HIF-1/BNIP3/Beclin1 signalling pathway modulates autophagy and contributes to hypoxia preconditioning-induced protection against OGD/Reperfusion. Although rapamycin promoted autophagy (shown by LC3-II upregulation and p62 downregulation), it did not upregulate the expression of Beclin1. This is in line with studies by Li *et al*.^36^ and Grishchuk *et al*.^37^, which found that rapamycin promoted autophagy via a Beclin1 independent mechanism. They showed that rapamycin inhibited mTOR resulting in activation of Atg1 kinase complex to regulate autophagy.

Autophagy is a self-catabolic process of damaged or dysfunctional cellular components, which are recycled for providing energy and nutrients^29^. A number of studies demonstrate that mild/moderate autophagy has a neuroprotective effect on ischaemic stroke whereas insufficient or excessive autophagy results in nerve damage and cell death^22,29^. A study by Park *et al*.^38^ showed ischaemic preconditioning (IPC) increased generation of autophagosomes and lysosomal activity. Blocking autophagic activity by 3-methyladenine during IPC ameliorated the neuroprotective effects of IPC, indicating that autophagy participates in IPC-induced neuroprotection. Similarly, Sheng *et al*.^39^ showed that autophagy activation during IPC plays an important role in developing tolerance against a subsequent fatal ischaemic insult. Yan *et al*.^40^ showed that elevated autophagic activity contributed to increased tolerance against transient focal cerebral ischaemia in hyperbaric oxygen preconditioning while inhibition of autophagy using 3-methyladenine supressed the protective effective. Additionally, pre- and post- conditioning with rapamycin were found neuroprotective in various *in-vivo* and *in-vitro* studies^22,41^.

At 100 μM, all the inhibitors except DMOG were able to accumulate HIF-1α significantly in the PC12 cells after 24 hours incubation at normoxia. We subsequently demonstrated, despite resulting in significant cytotoxicity DMOG at both 1 and 2 mM stabilized the HIF-1α in PC12 cells. This indicates that the 4 ‘clinical’ compounds have higher potency compared to DMOG for inducing HIF-1α in PC12 cells. This conclusion is consistent with studies by Zhdanov *et al*.^42^ in PC12 cells and by Chan *et al*.^19^, in MCF-7 cells, where 1 mM DMOG significantly stabilised HIF-1α. In the primary neurons, the HIF-1α was stabilised by FG4592 (30 μM) and DMOG (100 μM), whose concentrations were significantly lower compared to those used in PC 12 cells. FG4592 clinical trial concentration is 30 µM^43^. 50 µM of FG4592 was shown to upregulate HIF-1α expression in SH-SY5Y cells in normoxia^31^. In line with our results in primary rat neurons, Ogle *et al*.^9^, treated primary rat neurons with DMOG (up to 500 µM) for 24 hours at normoxia and showed increased levels of HIF-1α protein. Badawi and Shi^44^ treated primary rat neurons with 0.5, 1 and 2 mM DMOG and HIF-1α protein levels were significantly increased by 57%, 83% and 93% respectively. DMOG at 2 mM is close to it maximal effect as a higher concentration of DMOG did not cause further increase in the HIF-1α protein level.

Six hours OGD (0.3% O_2_), led to about 40% of cell death. Pre-treatment with the PHD inhibitors at 100 µM for 24 hours with 24 hours reoxygenation provided significant cytoprotection of both PC12 cells and primary neurons following 6 hours OGD. This is consistent with the study by Ogle *et al*.^9^ that preconditioning with DMOG (100 μM) for 24 hours prior to OGD significantly reduced OGD-induced neuron cytotoxicity. Jones *et al*.^45^ reported that pre-treatment with DMOG > 30 µM for 20 hours prior to OGD significantly reduced OGD-induced neuronal death in a dose-dependent manner. DMOG induced protection has also been demonstrated *in-vivo*, in which the systemic application of DMOG before onset of cerebral ischaemia and throughout led to increased acute cerebral tissue preservation^7^. Whilst, post-treatment with DMOG or FG4497 attenuated sensorimotor dysfunction in mice 3 days or 7 days after ischaemia-reperfusion injury^9,11,18^.

There are few studies on the 4 ‘clinical’ PHD inhibitors in stroke research. GSK1278863 (Daprodustat), a pyrimidinetrione-glycinamide, is at least partially, selective for the PHDs over other 2-OG dependent enzymes, which minimises off-target effects whilst also signifying an advantage over DMOG^5,46^. Another PHD inhibitor from GlaxoSmithKline, GSK360A has been shown to decrease the infarct volume and improve behaviour after transient MCAO in rats^12^. FG2216 (IOX3) is structurally related to FG4592, and was replaced in clinical development by FG4592 following the appearance of adverse effects in clinical trial phase II^24^. There is an additional phenoxy-group on the phenyl isoquinolyl ring in FG4592, resulting in more efficient binding than FG2216^5^. FG2216 offered neuroprotective when pre-treating mice followed MCAO^10^.

Our study reveals that 6 hours OGD mainly results in apoptosis, rather than necrosis in the PC 12 cells, consistent with the previous studies^47^. Pre-treatment with PHD inhibitors protected PC12 cells from apoptosis following OGD. These findings correlate with a finding of a study by Li *et al*.^34^, where FG4592 reduced MPP + (1-methyl-4-phenylpyridinium) induced apoptosis and improved mitochondrial function in SH-SY5Y cells. Apoptosis is energy-dependent, reversible programmed cell death that results in rapid clearance of cells by phagocytosis. HIF-1α stabilisation induces apoptosis by stabilising of tumour suppressor gene p53, which induces Bax and Bak proteins regulating the release of cytochrome C^48^. HIF-1α stabilisation also results in upregulation of proapoptotic family members such as BNIP3, Noxa, Nix and downregulation of Bcl-2^49^. By contrast, HIF-1α can also protect cells from apoptosis by elevating Bcl-2 and Mcl-1 levels, Bcl-xL induction, and decreasing pro-apoptotic Bid, Bax, and Bak levels^50^. In our study, the PHD inhibitors (100 µM) treatment for 24 hours stabilised HIF-1α, and protected the cells from a subsequent OGD injury.

*Hif1a* gene expression was not significantly altered by the PHD inhibitors in the PC 12 cells despite the HIF-1α protein levels being significantly increased by the PHD inhibitors. Our observations are consistent with various studies that indicate that HIF-1α levels are mainly regulated at the post-transcriptional level^51,52^. During ischaemia, cells switch from oxidative phosphorylation to anaerobic glycolysis. Significant upregulation of *Pfkfb3* was seen all the five PHD inhibitors, which is consistent with the study by Minchenko *et al*.^53^ that revealed cobalt chloride, DFO and DMOG stimulated Pfkfb3 mRNA expression in Hep3b cells. PFKFB3 is ubiquitously expressed in several proliferating cells and tissues^54^. No significant changes in *Pfkfb1* in the PC12 cells by the PHD inhibitors were seen in this study. FG2216, GSK1278863 and Bay 85–3934 significantly upregulated the *Ldha* expression in PC12 cells, while FG4592 and DMOG did not. HIF signalling possesses feedback loops, e.g. PHD2 and PHD3 are upregulated in a negative feedback loop with HIF-1 stabilisation^55^. The *Phd2* expression was significantly upregulated by FG4592 in PC12 cells. FG4592 and Bayer 85–3934 significantly upregulated *Bnip3* and *Vegf* expression. It has been recently discovered that the upregulation of BNIP3 and BNIP3L provides a protective effect through promoting mitochondrial autophagy via competing with Bcl-2 releasing Beclin-1^30^. VEGF plays an important role in stimulating angiogenesis and neurogenesis resulting in increased blood flow and metabolism. VEGF also promotes cell survival by promoting survival-promoting signalling pathways, such as serine-threonine protein kinase Akt signal transduction system and induction of anti-apoptotic pathways^56^. VEGF is reported to reduce caspase-3 activation and cell death in an *in-vitro* model of cerebral ischaemia^57^. Nevertheless, VEGF was shown to induce vascular permeability, resulting in oedema formation after stroke^56,57^.

In conclusion, the HIF PHD inhibitors possess the capability to stabilise HIF-1α and to upregulate expression of a number of hypoxia genes that promote ischaemic tolerance. The PHD inhibitors induce autophagy in both PC 12 cells and primary neurons, but do not cause apoptosis or cell death at 100 μM. DMOG is less potent in stabilising HIF-1α, autophagy induction and ischaemic tolerance compared to the ‘clinical’ PHD inhibitors. The pharmacological induced hypoxia adaptation requires coordination of intricate pathways and mechanisms, such as HIF, mTOR, autophagy. Further studies for precise understanding of the interplay of these mechanisms could lead to the development of new pharmacological strategies to minimize the damaging effects of strokes.

## Materials and Methods

### Materials

Rat adrenal pheochromocytoma (PC12) cells, Dulbecco’s Modified Eagle’s Medium (DMEM) containing high glucose (4.5 g/L), Dulbecco’s phosphate buffered saline (PBS), fetal bovine serum (FBS), inactivated horse serum (HS), poly-D-lysine(50x), trypsin(50x), Dimethyloxaloglycine (DMOG), rapamycin, 3-(4, 5-dimethylthiazol-2-yl)-2, 5-diphenyltetrazolium bromide (MTT), dimethyl sulfoxide (DMSO), poly-D-lysine(50x), Trypan Blue, protease inhibitor cocktail, phenylmethylsulfonyl fluoride (PMSF), Tween-20, sodium chloride, Tris, glycine, sodium-dodecyl sulphate (SDS), dithiothreitol (DTT), Triton X-100, paraformaldehyde (PFA), bovine serum albumin (BSA), goat anti-rabbit IgG- FITC, goat anti-mouse IgG- FITC antibody were from Sigma-Aldrich (St Louis, MO,USA).

Glucose-free Dulbecco’s modified eagle medium, Neurobasal medium, Glucose-free Neurobasal medium, penicillin and streptomycin (10000 units/mL & 10000 µg/mL), TrypLE (synthetic trypsin), Glutamax supplement, sodium pyruvate (100 mM), Hank’s Balanced Salt solution (HBSS), L-glutamine (200 mM), B27 supplement (50×, serum free), Pierce BCA protein assay kit and Pierce ECL western blotting substrates were from ThermoFisher Scientific (Loughborough, UK). Laemmli buffer(4x), 4–15% Mini-PROTEAN TGX Precast polyacrylamide gel, skimmed milk, Precision Plus Protein Dual Color Standard were from Bio-Rad (Hertfordshire, UK). Amersham™ Protran® Premium nitrocellulose blotting membranes were from VWR (Leicestershire, UK), RIPA (radio-immuno precipitation assay) buffer(10x) were from New England Biolabs Ltd (Hertfordshire, UK), Mouse anti- HIF-1α monoclonal antibody was from Novus Biologics (Abington, UK), rabbit anti-p62 (SQSTM1) polyclonal antibody, rabbit anti-LC3B polyclonal antibody, and rabbit polyclonal anti-β-actin antibody were from Abcam (Cambridge, UK), rabbit polyclonal anti-Beclin1 polyclonal antibody from Cell signalling technology (Danvers, MA, USA), goat polyclonal anti-mouse IgG HRP affinity, anti-rabbit IgG HRP affinity were from Dako, Agilent (Santa Clara, CA, USA). Monoclonal mouse anti-Tuj1 was obtained from Biolegend (San Diego, CA, USA). Vectashield mounting medium with DAPI was obtained from Vector Laboratories (Burlingame, CA, USA).

The Tetro cDNA synthesis kit and SensiFAST^TM^ SYBR Hi-ROX kits were from Bioline Reagents Ltd (London, UK). The RNeasy plus Mini Kit was from Qiagen (Manchester, UK). The non-radioactive cytotoxicity assay kits were from Promega (Southampton, UK). The Guava cell dispersal reagent, Guava nexin kit, Guava instrument cleaning fluid, Guava Easycheck kit were from Merck Millipore (Burlingon, MA, USA). Plastic materials for cell cultures including pipettes, t25 cell culture vessel, 96-, 24- and 12-well plates, were from Greiner Bio-One (Gloucestershire, UK).

The PHD inhibitors, FG2216 (IOX3), FG4592, GSK1278863 and Bay 85–3934 were prepared, as reported^5^.

### Cell culture

#### PC12 cells

PC12 cells were cultured in ‘complete’ medium (high-glucose DMEM (containing 4 g/L glucose, L-glutamine and sodium bicarbonate, without pyruvate) supplemented with 5% FBS, 5% HS and 1% penicillin-streptomycin. PC 12 cells were incubated at 37 °C in a humidified atmosphere of 5% CO_2_ in air (ESCO cell culture CO_2_ incubator, Barnsley, UK). The plates and flasks used were coated with 5 mg/mL poly-D-lysine if required.

#### Primary cortical rat neurons culture

Rat embryos E17–18 were removed from a pregnant rat, which was humanely killed under Schedule 1 according to the Animals Scientific Procedures Act (1986) (ASPA). The work is exempt from the need for Animal Welfare and Ethical Review Board (AWERB) approval under the ASPA and all subsequent amendments under both UK and European Law. All animals used in this study have been treated in accordance with ASPA guidelines. The embryonic brains were placed in HBSS on ice and dissected, and the cortices removed into an Eppendorf containing cold Neurobasal medium. The Neurobasal medium was then removed and replaced with pre-heated (37 °C) Neurobasal medium containing 0.05% trypsin and 100 µg/mL DNAse and the cortices dissociated through pipetting. When dissociated, the cortices were placed in a 15 mL falcon tube and placed in the incubator (37 °C) for 15 minutes. Thereafter, the cortices were removed and 6 mL pre-heated (37 °C) Neurobasal medium containing 10% FBS added in order to inactivate the trypsin. The cortices were further dissociated through pipetting before the cell suspension was centrifuged in a Harrier 18/80 R (MSE, East Sussex, UK) at 1500 rpm for 5 minutes. The supernatant was discarded, and the cells re-suspended in Neurobasal medium containing 2% B27 serum-free supplements, 2mM L-glutamine and 1% penicillin and streptomycin and filtered through a 70 micron filter. Neurons were then plated onto 5 mg/mL poly-D-lysine pre-coated plates at a density of approximately 0.15 × 10^6^ cells per cm^2^. Thereafter, plates were placed in a standard incubator with a humidified atmosphere containing 5% CO_2_ at 37 °C. Typically, experiments were performed at DIV 9–14.

### Procedure of oxygen and glucose deprivation (OGD)

To mimic cerebral ischaemia *in vitro*, combined oxygen and glucose deprivation (OGD) was implemented. For OGD, the ‘complete’ medium was aspirated, and PC 12 cells rinsed twice with glucose-free media before incubation in glucose-free DMEM. PC 12 cells were then transferred into a purpose-built INVIVO_2_ 400 humidified hypoxia workstation (Ruskinn Technologies, Bridgend, UK) with atmospheric conditions established at 0.3% O_2_, 5% CO_2_, 94% N_2_ and 37 °C for 6 hours. For OGD in primary cortical neurons, neurons were switched to glucose-free Neurobasal medium containing 2% B27 serum-free supplements, 2mM L-glutamine and 1% penicillin and streptomycin. All other experimental parameters remained constant with PC12 cells.

### Drug administration

The PHD inhibitors (DMOG, FG2216, FG4592, GSK1278863, Bay 85–3934) and an autophagy inducer (rapamycin) were initially dissolved in DMSO and subsequently diluted in the treatment appropriate culture medium to the indicated concentrations. To analyse the cytoprotective effects of the PHD inhibitors, final concentrations of 1, 10, 50 or 100 µM were applied in PC12 cells. The effect of rapamycin was studied at a final concentration of 1 and 10 µM. In primary rat cortical neurons, the PHD inhibitors DMOG and FG4592 were studied at varying concentrations as indicated. For the vehicle control group, a final concentration of 1% (v/v) aqueous DMSO was used throughout. To study the cytotoxicity of PHD inhibitors initial experiments involved exposure for 24 h to varying concentration during normoxia in ‘complete’ medium.

The cytoprotective effects of the PHD inhibitors were tested in an ischaemic tolerance model^25^. The cells were pre-treated with the PHD inhibitors for 24 hours, were then subjected to a period of reoxygenation (24 hours) by rapidly replacing the medium with normal ‘complete’ media and returning cells to normoxia, thereafter the cells were treated with an OGD (0.3% O_2_) insult for 6 hours.

### Assessment of cell viability

#### MTT assays

Cell viability was evaluated using the standard colorimetric assay for mitochondrial reductase catalysed reduction of yellow MTT to give a purple formazan product. PC12 cells were seeded on poly-D-lysine pre-coated 96-well plates at a density of 1.2 × 10^4^ cells/well and cultured in ‘complete’ medium. Four hours prior to the completion of the experimental treatment, 10 μL of 5 mg/mL MTT solution diluted in PBS was added to the culture medium (final concentration, 0.5 mg/mL), and all samples incubated at 37 °C under treatment conditions. On completion of treatment, the supernatant was aspirated and the formazan crystals formed by surviving cells were solubilised in 50 μL DMSO, then incubated at 37 °C for 10 minutes. The optical density (OD) value of each well was determined by reading the absorbance at 540 nm using a microplate reader (Tecan Infinite M200 PRO, Switzerland). The DMSO background was subtracted from absorbance readings and the viability of cells for each treatment group was calculated based on Eq. 1;

1 $$MTT\,( \% \,of\,control)=\frac{OD\,value\,(Experimental\,group}{OD\,value\,(control\,group)}\times 100 \% $$

The viability of control cells (complete media in normoxic conditions) were assigned as 100% viable, while treatment samples were normalised against the control group OD value. Results are expressed as the percentage of cells possessing the ability to reduce MTT.

#### Lactate dehydrogenase (LDH) release assay

Cell-membrane integrity was analysed by measuring LDH activity of the culture medium using a non-radioactive cytotoxicity assay kit. PC12 cells were seeded on poly-D-lysine pre-coated 96-well plates at a density of 1.2 × 10^4^ cells/well and cultured in ‘complete’ medium. Maximum LDH release control was generated by adding 10 µL 10x lysis solution to wells containing control cells, 45 minutes prior to adding the CytoTox reaction mixture. Following the completion of the experimental treatment, 50 µL of each sample medium was transferred to an unused 96-well flat bottom plate, into which, a further 50 µL of the reaction mixture containing tetrazolium salts was added. The subsequent mixture was incubated at room temperature for 30 minutes, protected from light. After 30 minutes, 50 µL of stop solution was added to each well and mixed. The amounts of formazan dye formed were assessed by measuring the absorbance with a microplate reader at 490 nm. The absorbance of the culture media background was subtracted from experimental values and the percent cytotoxicity calculated using Eq. 2:

2 $$Percent\,Cytotoxicity\,( \% )=100\times \frac{Experimental\,LDH\,Release}{Maximum\,LDH\,Release}$$

The data are expressed as the mean percent of LDH release from the maximum control.

#### Trypan blue exclusion assay

Trypan blue exclusion was used to determine viable cells present in cell suspensions. This assay is based on the principle that live cells possess intact cell membranes and exclude trypan blue dye. PC12 cells were seeded at density of 1.2×10^6^ cells in T25 and incubated in the complete DMEM media. Following treatments, the cell suspension was transferred in 15 ml sterile centrifuge tube and centrifuged for 5 minutes (100 relative centrifugal force (rcf)) using a Harrier 18/80 R centrifuge (MSE, London, UK). The medium was aspirated and the cells were re-suspended in complete DMEM media. Cells clusters were mechanically broken by pipetting. 100 μL of the suspension was stained with 100 μL 0.4% trypan blue for 5 minutes. 10 μL of the trypan-blue treated cell suspension was applied to each side of the haemocytometer. Live cells possessing a clear cytoplasm and non-viable cells possessing a blue cytoplasm were counted in each culture. Five samples were analysed for each treatment condition.

3 $$ \% \,live\,cells=\frac{Viable\,cells}{Total\,number\,of\,cells\,(Viable+dead)}\times 100 \% $$

Cell viability was expressed as percentage of viable cells in the total number of cells based on the Eq. 3.

### Flow cytometry analysis

A Guava Nexin Kit containing Annexin V and 7-AAD double stain was used to assess early apoptosis. Annexin V is a calcium-dependent phospholipid binding protein with affinity for phosphatidylserine (PS), a membrane component normally localized to the internal face of the cell membrane. Early apoptosis results in translocation of PS molecules to outer surface of cell membrane where Annexin V can bind to them. 7-AAD is excluded in late- apoptotic and dead cells. PC12 cells were plated at a density of 1 × 10^6^ in T25 flasks and grown overnight at 37 °C. At completion of identified treatment, 100 μl of cell suspension was transferred into 1.5 ml microcentrifuge tube. To break cells clusters, 50 μl 0.8x Guava cell dispersal reagent was added to the tube and incubated for 20 minutes at 37 °C. The cell suspension was microcentrifuged for 5 minutes (14,500 g) and the culture medium was aspirated. 100 μl of DMEM and 100 μl of the Guava Nexin reagent were then added. The sample was stained for 20 minutes at room temperature, and were analysed using a Guava easyCyte flow cytometer (Merck Millipore, MA, USA). Data were analysed using Guava analysis software (Merck Millipore, MA, USA). A total of 10000 events in the gate were acquired for each sample and three samples were acquired per condition. The data were expressed as percentage of cells in each quadrant. Cells in lower left quadrant represented viable cells (Annexin V and 7-AAD negative cells); the cells in lower right quadrant represent early apoptotic cells (Annexin V positive); cells in upper right column represent necrotic/late apoptotic cells (Annexin V and 7-AAD positive cells).

### Immunofluorescence

Cell were fixed with 4% paraformaldehyde (PFA) for 15 minutes, and then were permeabilised using 0.1% Triton X-100 in PBS for 15 minutes and blocked by incubating with 5% bovine serum albumin (BSA) in PBS-T (PBS + 0.1% Triton X-100) for 1 hour in room temperature. This was followed by overnight incubation at 4 °C with primary antibody (mouse anti-Tuj1, 1:1000 in 1% BSA in PBS-T). Following three PBS washes, cells were incubated in secondary antibody (goat anti-mouse IgG-FITC, 1:200 in 1% BSA in PBS-T) for 2 hours at room temperature. Coverslips were then washed with PBS and mounted onto slides with Vectashield mounting medium with nuclear stain DAPI (4′, 6-diamidino-2-phenylindole). Images were taken by Nikon Eclipse 80i fluorescence microscope (Japan), Hamamatsu (C4742-95) digital camera and were double merged (consisting of FITC Tuj1;^+^ DAPI stained nuclei) with NIS-Element BR 3.22.14 software (Nikon, Tokyo, Japan).

### Protein extraction and analysis

Cells were plated at a density of 1 × 10^6^ in T25 flasks. After experimental treatments, the cells were washed with ice-cold PBS. Protein extraction buffer was made using 5 ml 1xRIPA buffer, 1 mM PMSF (50 μL) and a protease inhibitor cocktail (50 μL). Ice cold lysis buffer (0.5 ml per 75 cm^2^) was added and mixed on orbital shaker for 15 minutes. Adherent cells were scraped using plastic cold scraper and transferred to an ice-cooled microcentrifuge tube. The sample was sonicated (3 cycles of 10 seconds pulse with 5 seconds interval) (MSE Soniprep 150 Plus, Wolflabs, UK), and centrifuged for 10 minutes. The resulting supernatant was transferred to a new microcentrifuge tube and the protein concentrations were determined using a BCA protein assay kit following the manufacturer’s instructions.

Twenty to 40 µg protein was denatured for 5 minutes in Laemmelli buffer at 95 °C. Samples were loaded on to pre-cast 4–20% polyacrylamide miniPROTEAN TGX gels (10 wells). 10 µl of Precision plus ProteinTM standards (10–250 kD) was loaded in each gel. The gels were run in 1x Tris/glycine/SDS running buffer in a BioRad Mini-PROTEAN® tetra cell at 100 volts connected to a BioRad PowerPac Basic until the blue dye reached bottom (black line) of the gel. Following which the gels were removed from their cassettes into 1x transfer buffer. A trans-Blot SD Semi-Dry Electrophoretic transfer cell (Bio-Rad, Hertfordshire, UK) was used for the transfer. Gels were placed onto a nitrocellulose membrane and assembled with extra-thick filter paper. Transfer was performed at 12 volts for 1 hour. Membranes were blocked with 5% milk powder in 1x PBS-T for 1 hour then incubated overnight at 4 °C with primary antibodies against anti- HIF-1α (1:500), anti-Lc3b (1:500), anti-Beclin1 (1:500), anti-p62 (1:500) which were prepared in 1% milk powder in 1x PBS-T buffer. After the overnight incubation, membranes were washed in 1x PBS-T three times for 5 minutes each and were incubated for 1 hour in a horseradish peroxidise (HRP) conjugated secondary antibody, made in 1% milk powder in 1x PBS-T. After being washed three times with 1xPBS-T, the membranes were developed by Pierce ECL Western immunoblotting substrates, and imaged using FluorChem Western Blot imaging system (ProteinSimple, CA, USA) or ChemiDoc MP Imaging system (Biorad, UK). Thereafter, the membranes were stripped with mild-stripper buffer and re-probed with anti β-actin antibody (1:1000) and a subsequent HRP conjugated secondary antibody. The protein levels were quantified by densitometric analysis using Image J (NIH, USA). Values were normalized to β-actin and the corresponding controls.

### Quantitative real-time polymerase chain reaction (qRT-PCR)

RNA was extracted from cells using the RNeasy plus Mini Kit. The cell pellet was harvested by transferring the cell suspension to 15 mL centrifuge tube and centrifuging (5 minutes, 100 rcf). After which the supernatant was removed and 350 μL RLT plus containing 40 μM DTT was added to lyse the cell pellet. The homogenised lysate was then transferred to the supplied gDNA Eliminator spin column. The flow through was mixed with equal volume of 70% ethanol and transferred to an RNeasy spin column. The RNA pellets were washed with RW1 and RPE buffer following the manufacturer’s instruction. The resultant RNA pellets were eluted in 50 μL nuclease-free water and the concentration measured on the NanoDrop 1000 Spectrophotometer (ThermoFisher Scientific, Loughborough, UK). The quality of the RNA samples was assessed using the 260/280 and 260/230 absorbance measurements. After extraction, each sample was diluted to a total RNA concentration 2 μg and transferred to a 1.5 mL RNase-free microcentrifuge tube on ice, ready for cDNA synthesis. Using Oligo(dT)_18_ primers cDNA was synthesised using the Tetro cDNA synthesis kit in accordance with the manufacturer’s protocol. Amplification of 100 ng cDNA template per reaction was performed using a Techne PROPLATE48, using the SensiFAST SYBR Hi-ROX kit. Using a Techne Prime Pro 48 Real-time qPCR machine (ThermoFisher Scientific, Loughborough, UK), an initial activation step was performed for 2 minutes at 95 °C before 40 cycles of a 3-step cycling program consisting of 95 °C for 5 seconds, 60 °C for 10 seconds and 72 °C for 15 seconds. The primers of a number of hypoxia genes [hypoxia-inducible factor 1α (*Hif1α*), BCL2/adenovirus E1B 19 kDa protein-interacting protein 3 (*Bnip3*), HIF target gene prolyl hydroxylase 2 (*Phd2*), vascular endothelial growth factor (*Vegf*), 6-phosphofructo-2-kinase/fructose-2,6-biphosphatase 1 (*Pfkfb1*), 6-phosphofructo-2-kinase/fructose-2,6-biphosphatase 3 (*Pfkfb3*), lactate dehydrogenase A (*Ldha*)] used for analysis are in Table 1. *Actin* was used as a house keeping gene to normalise the relative levels of mRNA. β-Actin has been used as an internal loading control in PC12 cells for hypoxia treatment in previous studies^58,59^. Quantification of mRNA expression was performed using the comparative delta Ct method.

**Table 1:** List of primers used for qRT-PCR studies and their forward (FW)/reverse (RV) sequences.

*HIF1α*	FW TCAAGTCAGCAACGTGGAAG RV TATCGAGGCTGTGTCGACTG
*Bnip3*	FW TTTAAACACCCGAAGCGCACAG RV GTTGTCAGACGCCTTCCAATGTAGA
*Phd2*	FW TGCATACGCCACAAGGTACG RV GTAGGTGACGCGGGTACTGC
*Vegf*	FW TTACTGCTGTACCTCCAC RV ACAGGACGGCTTGAAGATA
*Pfkfb1*	FW AACCGCAACATGACCTTCCT RV CAACACAGAGGCCCAGCTTA
*Pfkfb3*	FW CTGTCCAGCAGAGGCAAGAA RV CGCGGTCTGGATGGTACTTT
*Ldh-a*	FW AAGGTTATGGCTCCCTTGGC RV TAGTGACGTGTGACAGTGCC
*Actin*	FW TGCCCTAGACTTCGAGCAAGA RV CATGGATGCCACAGGATTCCATAC

### Data analysis

Experiments employing on the 96 well plate reader were performed with 3–8 well replicates. Independent experiments were performed in triplicate. The data is expressed as a mean value ± standard deviation (S.D.). One-way ANOVA with Tukey multiple comparison post-hoc was used to analyse comparisons among multiple groups. GraphPad PRISM 7 for Windows version 7.04 (GraphPad Software, Inc., CA, USA) was used for the analysis. Values of P < 0.05 were considered statistically significant.

## Supplementary information

Supplementary information.
