# Author Correction: Hypoxia-inducible factor (HIF) prolyl hydroxylase inhibitors induce autophagy and have a protective effect in an *in-vitro* ischaemia model

**DOI:** 10.1038/s41598-020-63108-2

**Published:** 2020-04-08

**Authors:** Ayesha Singh, James W. Wilson, Christopher J. Schofield, Ruoli Chen

**Affiliations:** 1 Institute for Science and Technology in Medicine, School of Pharmacy, Keele University, Newcastle under Lyme, Staffordshire, ST5 5BG United Kingdom; 2 Chemistry Research Laboratory, Department of Chemistry, University of Oxford, Oxford, OX1 5JJ United Kingdom

Correction to: *Scientific Reports* 10.1038/s41598-020-58482-w, published online 31 January 2020

James W. Wilson was omitted from the author list in the original version of this Article. This has been corrected in the PDF and HTML versions of the Article, and in the accompanying Supplementary Information file.

The Author Contributions section now reads:

This project was conceived by R.C. and C.J.S. The experiments in this paper were designed and performed by A.S., J.W.W. and R.C. The HIF PHD inhibitors were generated by C.J.S. Data were analysed and interpreted by A.S., J.W.W. and R.C. The manuscript was prepared by A.S and J.W.W. and commented by R.C and C.J.S. All authors have read and approved the final copy of the manuscript.

