# Supplementary material for: Hypoxia-inducible factor (HIF) prolyl hydroxylase inhibitors induce autophagy and have a protective effect in an in-vitro ischaemia model
Source: Sci Rep. 2020 Jan 31;10:1597. doi: 10.1038/s41598-020-58482-w (PMC6994562; doi:10.1038/s41598-020-58482-w)
Supplement: Supplementary file 1 — Supplementary information. [file 41598_2020_58482_MOESM1_ESM.pdf]

Hypoxia-inducible factor (HIF) prolyl hydroxylase inhibitors induce autophagy and have a protective effect in an *in-vitro* ischaemia model

Supplementary data

Ayesha Singh[1], James W Wilson[1], Christopher J. Schofield[2], Ruoli Chen[1*]

[1]Institute for Science and Technology in Medicine, School of Pharmacy, Keele University, Newcastle under Lyme, Staffordshire ST5 5BG, United Kingdom.

[2]Chemistry Research Laboratory, Department of Chemistry, University of Oxford, Oxford, OX1 5JJ, United Kingdom.

*** Correspondence to:**

Dr Ruoli Chen

[1]Institute for Science and Technology in Medicine,

School of Pharmacy,

Keele University,

Newcastle under Lyme,

Staffordshire ST5 5BG,

United Kingdom.

email: r.chen@keele.ac.uk

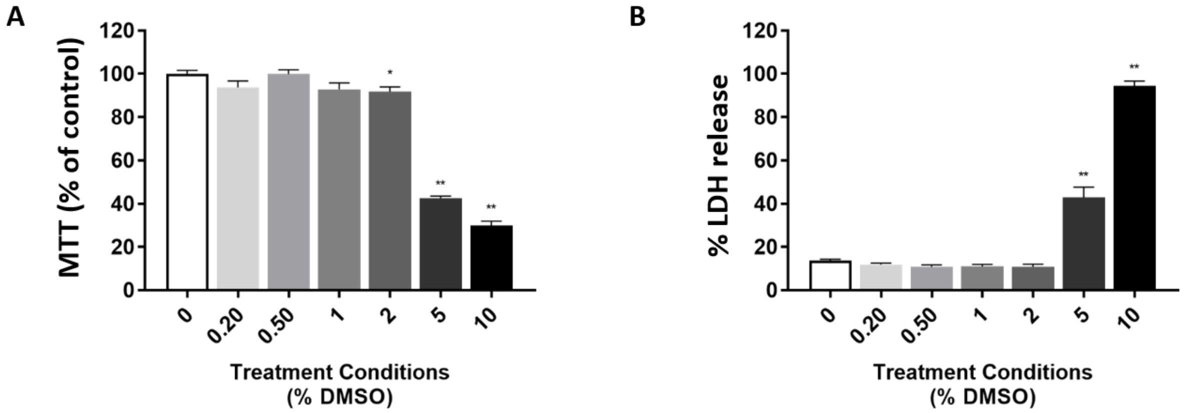

Fig. S1. Effects of DMSO on PC12 cell viability (MTT activity) and cytotoxicity (LDH release). PC12 cells were treated for 24 hours with the indicated DMSO concentrations. **(A)** Significant reduction in MTT activity was seen by 2%, 5% and 10% DMSO; **(B)** Significant increase in LDH release was seen by 5% and 10% DMSO. Results are expressed as means $\pm$ S.E.M from 3 independent experiments with statistical significance determined by one-way ANOVA Dunnett's test; $^{*}P<0.05$, $^{**}P<0.05$ *versus* time-matched control (Cntl; 21% O_2, normoxia) treated PC12 cells.

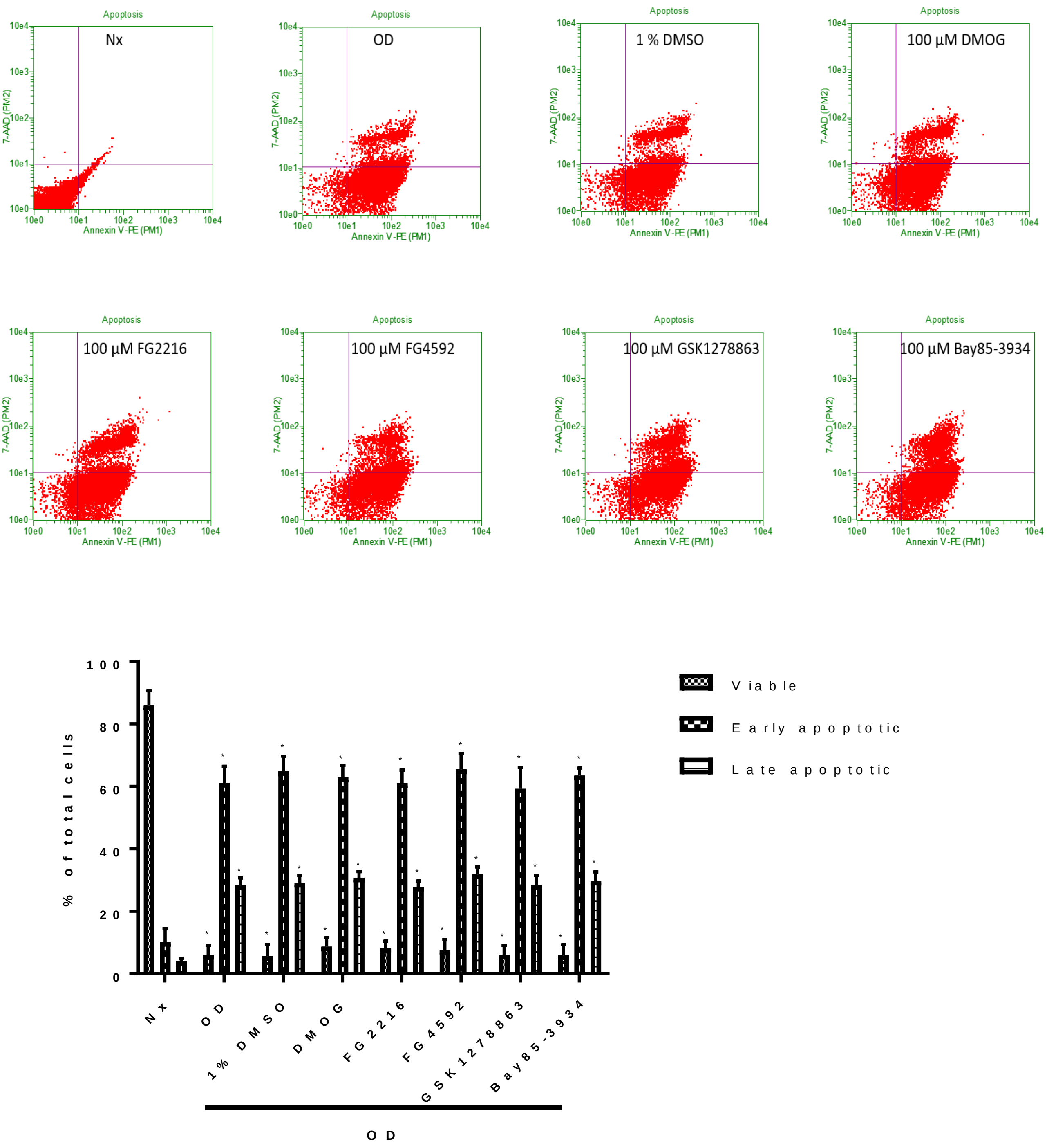

Figure S2. Annexin-V and 7-AAD FACS analysis of PC12 cells treated in normoxia, oxygen-deprivation (hypoxia, OD), or HIF-PHD inhibitors in hypoxia (OD) for 24 hours. PC12 cells were cultured and incubated 24 hours in normoxia (21% O_2), OD (0.3% O_2) with the PHD inhibitors (100 µM) as a positive control for apoptotic cell death (A) Representative dot plots of Annexin-V/7-AAD FACS analysis of cells treated with the vehicle (1% DMSO) and other indicated conditions. Cells in lower left quadrant represent viable cells, cells in lower right quadrant represent early apoptosis and cells in upper right quadrant represented late apoptosis/necrosis; (B) The group data (n=3) representing % of

viable, early and late apoptotic cells. In comparison to normoxia (Nx), there was significant increase in apoptotic cells in the OD with or without DMSO/PHD inhibitors. No significant difference in apoptosis was seen in the cells treated with OD only compared to those treated with PHD inhibitors in the OD, indicating that the PHD inhibitors didn’t induce apoptosis. Data are expressed as mean ± S.D.

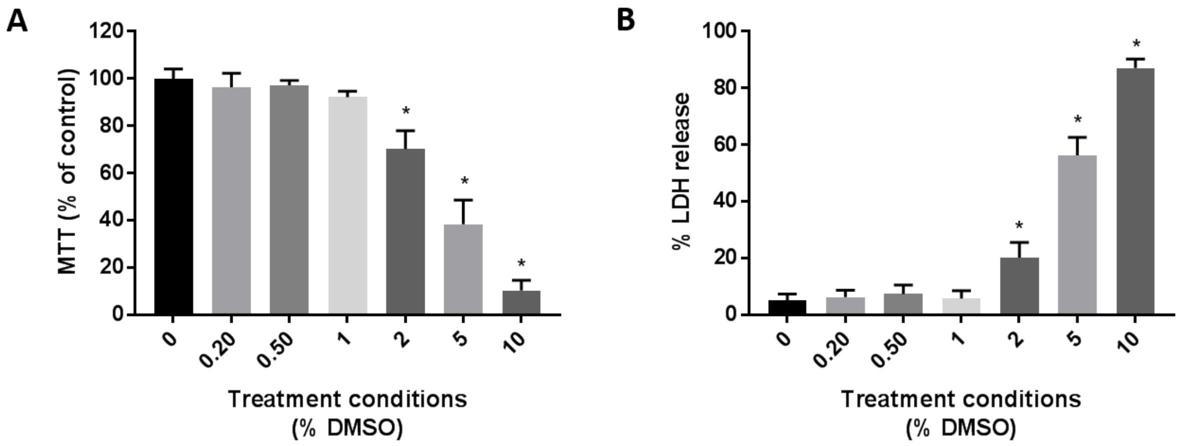

Fig. S3. Effects of DMSO on primary rat neurons viability (MTT activity) and cytotoxicity (LDH release). Primary rat cortical neurons were treated for 24 hours with the indicated DMSO concentrations. **(A)** Significant reduction in MTT activity was seen by 2%, 5% and 10% DMSO; **(B)** Significant increase in LDH release was seen by 2%, 5% and 10 % DMSO. Results are expressed as means ± S.D. from 3 independent experiments with statistical significance determined by one-way ANOVA Dunnett’s test; $^*P<0.05$ *versus* time-matched control (Cntl; 21% O_2, normoxia) treated primary neurons.

A

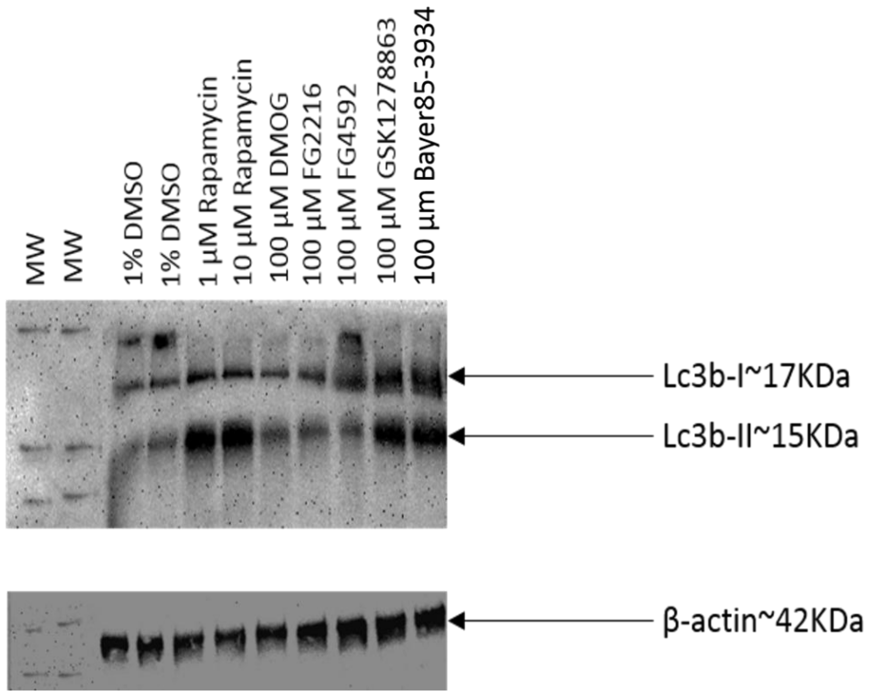

MW
MW
1% DMSO
1% DMSO
1 µM Rapamycin
10 µM Rapamycin
100 µM DMOG
100 µM FG2216
100 µM FG4592
100 µM GSK1278863
100 µm Bayer85-3934
Lc3b-I~17KDa
Lc3b-II~15KDa
β-actin~42KDa

B

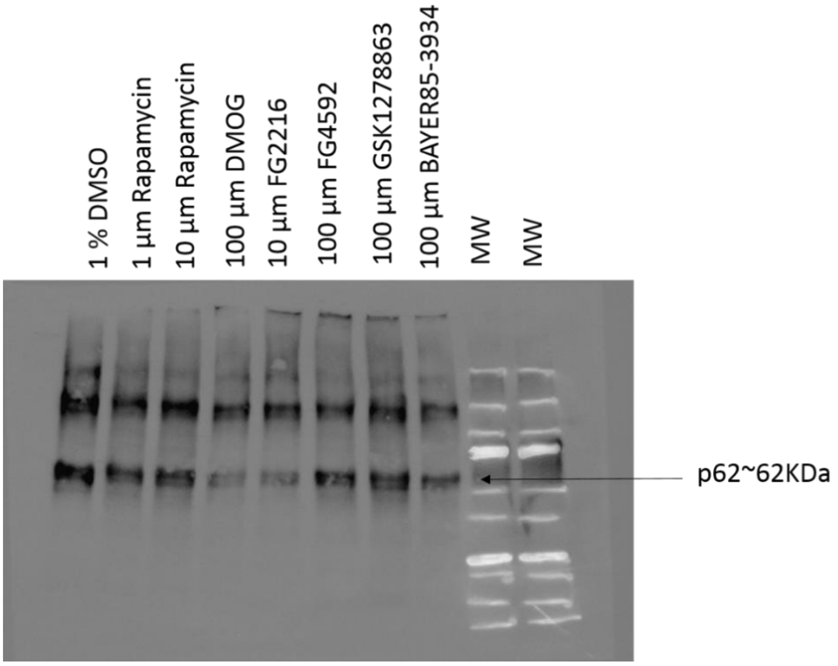

1 % DMSO
1 µm Rapamycin
10 µm Rapamycin
100 µm DMOG
10 µm FG2216
100 µm FG4592
100 µm GSK1278863
100 µm BAYER85-3934
MW
MW
p62~62KDa

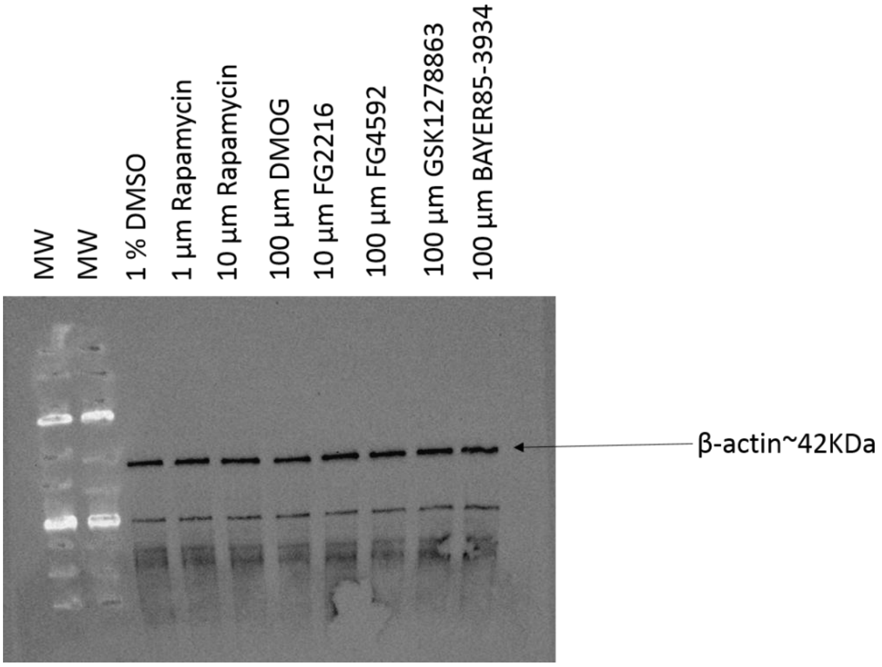

MW
MW
1 % DMSO
1 µm Rapamycin
10 µm Rapamycin
100 µm DMOG
10 µm FG2216
100 µm FG4592
100 µm GSK1278863
100 µm BAYER85-3934
β-actin~42KDa

C

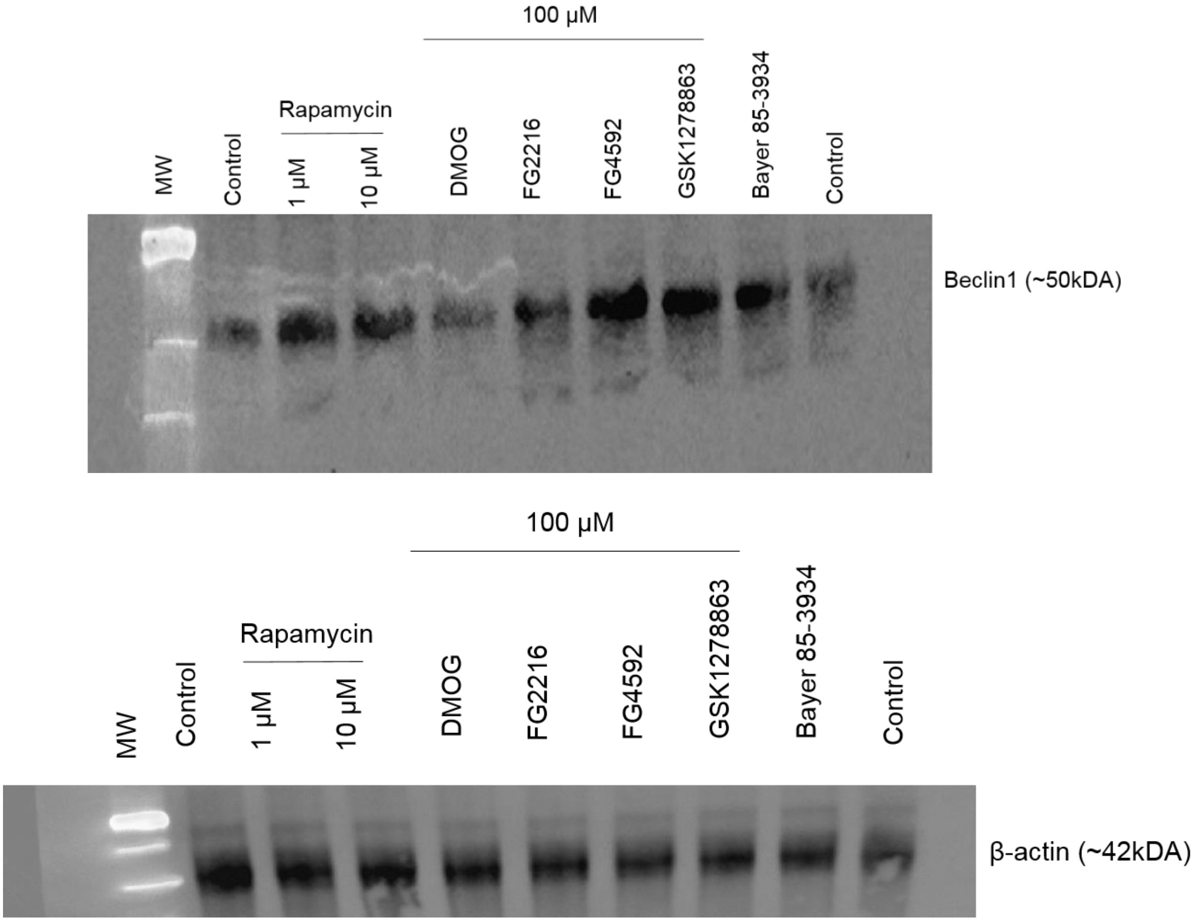

Figure S4. Effect of HIF-PHD inhibitors (100 µM) and Rapamycin (1 & 10 µM) on the Lc3b-II/Lc3b-I ratio, p62, Beclin1 in PC12 cells. (A) Immunoblot of the Lc3b-II/Lc3b-I ratio and corresponding β-actin in PC12 cells treatment with 24 hours of 100 µM of the indicated PHD inhibitors; 1 & 10 µM Rapamycin for 24 hours in normoxia; (B) Immunoblot of the p62 and corresponding β-actin in PC12 cells treatment with 24 hours of 100 µM of the indicated PHD inhibitors; 1 & 10 µM Rapamycin for 24 hours in normoxia; (C) Immunoblot of the Beclin1 and corresponding β-actin in PC12 cells treatment with 24 hours of 100 µM of the indicated PHD inhibitors; 1 & 10 µM Rapamycin for 24 hours in normoxia (MW represent molecular weight standard).

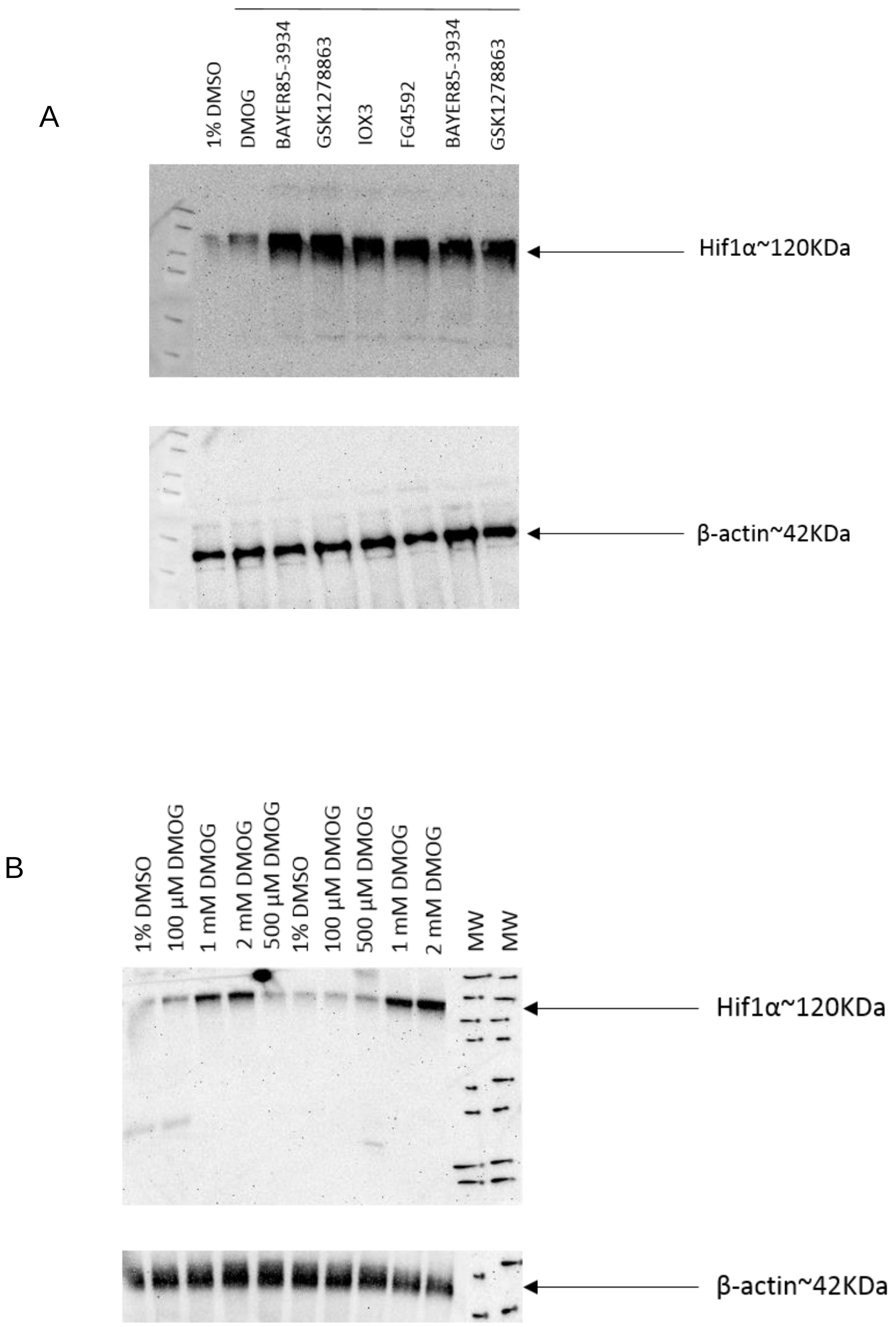

Figure S5. Effects of HIF-PHD inhibitors on HIF-1α levels in PC12 cells. (A) Representative HIF-1α were shown with those for β-actin treated with 100 µM of the indicated PHD inhibitors and 1% DMSO for 24 hours in normoxia (21% O_2); (B) Representative HIF1α immunoblots were shown with those for β-actin treated with 100 µM, 500 µM, 1 mM, 2mM of DMOG and 1% DMSO for 24 hours in normoxia (21% O_2).

A

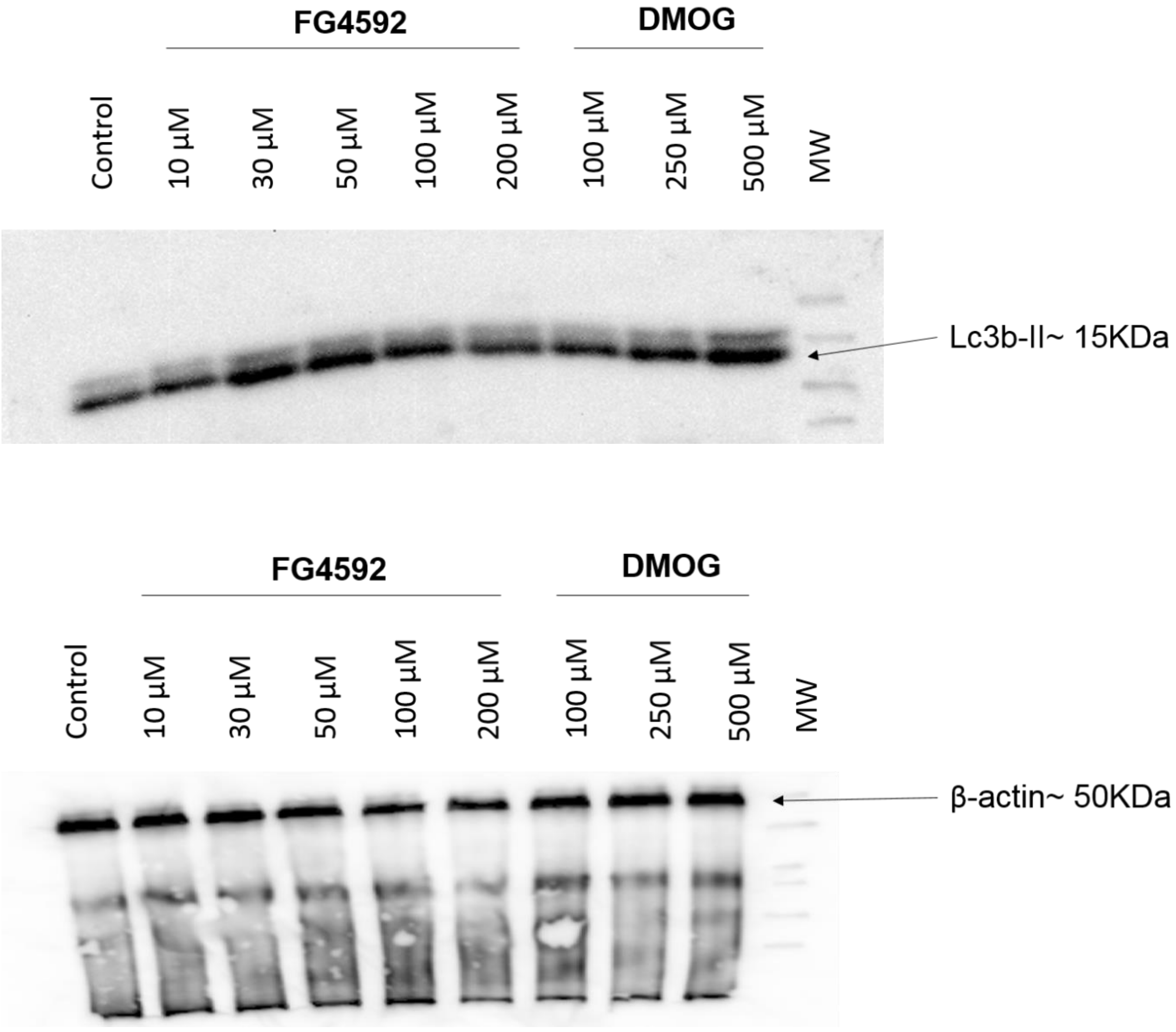
FG4592
DMOG
Control
10 µM
30 µM
50 µM
100 µM
200 µM
100 µM
250 µM
500 µM
MW
Lc3b-II~ 15KDa
FG4592
DMOG
Control
10 µM
30 µM
50 µM
100 µM
200 µM
100 µM
250 µM
500 µM
MW
β-actin~ 50KDa

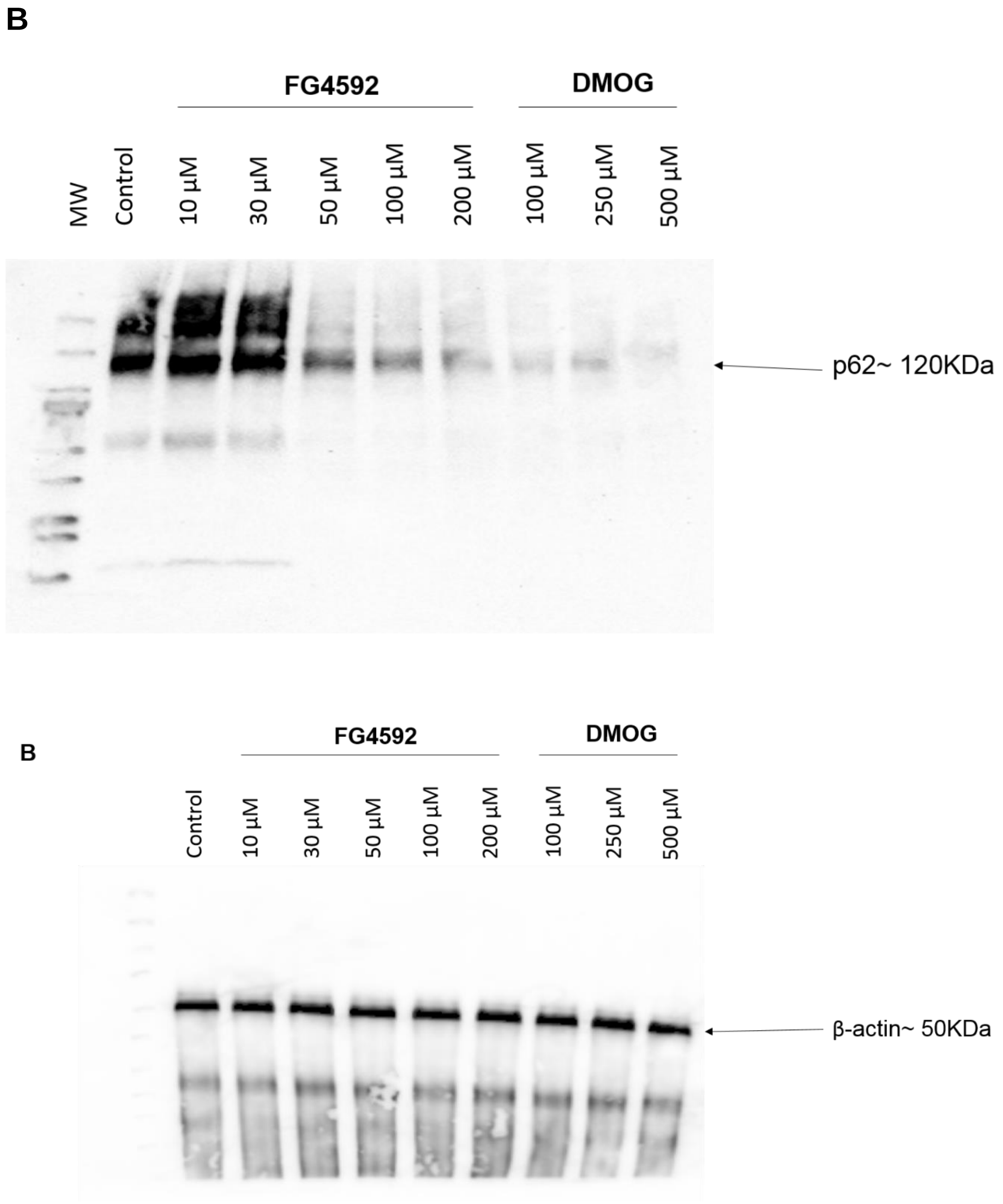

Figure S6. Effect of HIF-PHD inhibitors on the Lc3b-II/Lc3b-I ratio, p62 in primary rat neurons (A) Immunoblot of the Lc3b-II/Lc3b-I ratio and corresponding β-actin in primary cortical rat neurons treated with control (1% DMSO), 10,30, 50, 100 and 200 µM of FG4592 and 100, 250, 500 µM of DMOG for 24 hours in normoxia (21% O_2) (B) Immunoblot of the p62 and corresponding β-actin in primary cortical rat neurons treated with control (1% DMSO), 10,30, 50, 100 and 200 µM of FG4592 and 100, 250, 500 µM of DMOG for 24 hours in normoxia (21% O_2) (MW represent molecular weight standard).

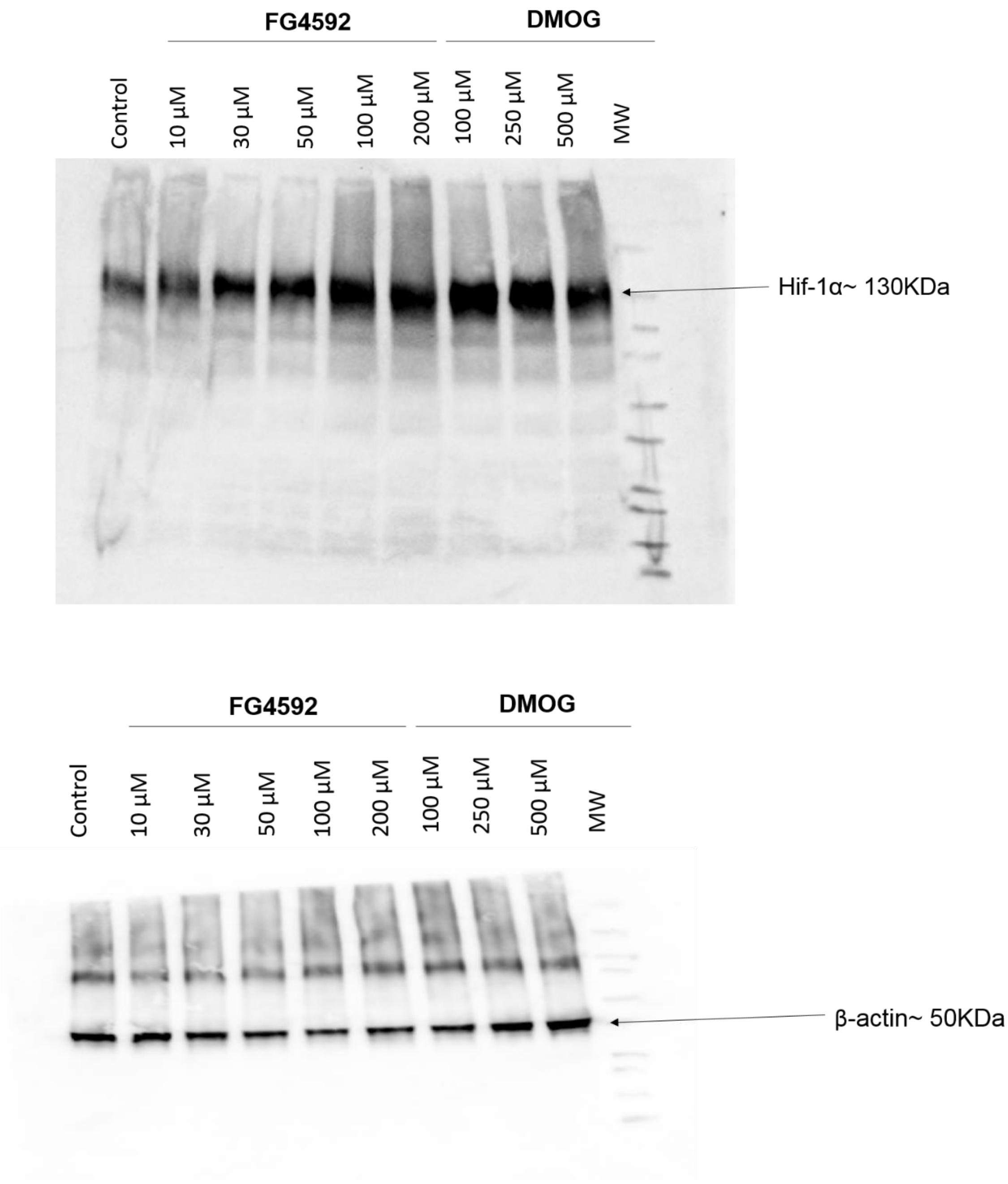

Figure S7. Effects of HIF-PHD inhibitors on HIF-1α levels in primary rat neurons. Representative HIF-1α were shown with those for β-actin treated with control (1% DMSO), 10, 30, 50, 100 and 200 µM of FG4592 and 100, 250, 500 µM of DMOG for 24 hours in normoxia (21% O_2), MW represents molecular weight standard
